# Distance-based clustering challenges for unbiased benchmarking studies

**DOI:** 10.1038/s41598-021-98126-1

**Published:** 2021-09-23

**Authors:** Michael C. Thrun

**Affiliations:** 1 DataBionics AG, Mathematics and Computer Science, The University of Marburg, Hans-Meerwein Str, 35032 Marburg, Germany; 2 Department of Hematology, Oncology and Immunology, Philipps-Universität Marburg, Hans-Meerwein-Straße 6, 04A28, 35032 Marburg, Germany

**Keywords:** Cancer, Computational biology and bioinformatics, Mathematics and computing

## Abstract

Benchmark datasets with predefined cluster structures and high-dimensional biomedical datasets outline the challenges of cluster analysis: clustering algorithms are limited in their clustering ability in the presence of clusters defining distance-based structures resulting in a biased clustering solution. Data sets might not have cluster structures. Clustering yields arbitrary labels and often depends on the trial, leading to varying results. Moreover, recent research indicated that all partition comparison measures can yield the same results for different clustering solutions. Consequently, algorithm selection and parameter optimization by unsupervised quality measures (QM) are always biased and misleading. Only if the predefined structures happen to meet the particular clustering criterion and QM, can the clusters be recovered. Results are presented based on 41 open-source algorithms which are particularly useful in biomedical scenarios. Furthermore, comparative analysis with mirrored density plots provides a significantly more detailed benchmark than that with the typically used box plots or violin plots.

Modern biomedical analysis techniques such as next-generation sequencing (NGS) have opened the door for complex high-dimensional data acquisition in medicine. For example, The Cancer Genome Atlas (TCGA) project provides open-source cancer data for a worldwide community. The availability of such rich data sources, which enable discovering new insights into disease-related genetic mechanisms, is challenging for data analysts. Genome- or transcriptome-wide association studies may reveal novel disease-related genes, e.g.^1^, and virtual karyotyping by NGS-based low-coverage whole-genome sequencing may replace the conventional karyotyping technique 130 years after von Waldeyer described human chromosomes^2^. However, deciphering previously unknown relations and hierarchies in high-dimensional biological datasets remains a challenge for knowledge discovery, meaning that the identification of valid, novel, potentially useful, and ultimately understandable patterns in data (e.g.,^3^) is a difficult task. A common first step is identifying clusters of objects that are likely to be functionally related or interact^4^, which has provoked debates about the most suitable clustering approaches. However, the definition of a cluster remains a matter of ongoing discussion^5,6^. Therefore, clustering is restricted here to the task of separating data into similar groups (c.f.^7,8^). Vividly, relative relationships between high-dimensional data points are of interest to build up structures in data that a cluster analysis can identify. Therefore, it remains essential to evaluate the results of clustering algorithms and grasp the differences in the structures they can catch. Recent research on cluster analysis conveys the message that relevant and possibly prior unknown relationships in high-dimensional biological datasets can be discovered by employing optimization procedures and automatic pipelines for either benchmarking or algorithm selection (e.g.,^4,9^). The state-of-the-art approach is to use one or more unsupervised indices for automatic evaluation, e.g., Wiwie et al.^4^ suggest the following guidelines for biomedical data:

"Use […] [hierarchical clustering*] or PAM. (2) Compute the silhouette values for clustering results using a broad range of parameter set variations. (3) Pick the result for the parameter set yielding the highest silhouette value" (*Restricted to UPGMA or average linking, see https://clusteval.sdu.dk/1/programs).

Alternatively, the authors provide the possibility of using the internal quality measures of Davies–Bouldin^10^ and Dunn^11^ indices.

The main contribution is to outline the pitfalls and challenges of approaches in which relevant and possibly prior unknown relationships in high-dimensional biological datasets are to be discovered by employing optimization procedures and automatic pipelines; more precisely, this work shows that.

- Parameter optimization on datasets without distance-based clusters,
- Algorithm selection by unsupervised quality measures on biomedical data, and
- Benchmarking clustering algorithms with first-order statistics or box plots or a small number of trials

are biased and often not recommended. Evidence for these pitfalls in cluster analysis is provided through the systematic and unbiased evaluation of 41 open-source clustering algorithms with several bodies of data that possess clearly defined structures. These insights are particularly useful for knowledge discovery in biomedical scenarios. Select distance-based structures are consistently defined in artificial samples of data with specific pitfalls for clustering algorithms. Moreover, two natural datasets with investigated cluster structures are employed, and it is shown that the data reflect a true and valid empirical biomedical entity.

This work shows that the limitations of clustering methods induced by their clustering criterion cannot be overcome by optimizing the algorithm parameters with a global criterion because such optimization can only reduce the variance but not the intrinsic bias.

This limitation is outlined in two examples in which, by optimizing the quality measure of the Davies–Boulding index^10^, Dunn index^11^ or Silhouette value^12^, a specific cluster structure is imposed, but the clinically relevant cluster structures are not reproduced. The biases of conventional clustering algorithms are investigated on five artificially defined data structures and two high-dimensional datasets. Furthermore, a clustering algorithm's parameters can still be significantly optimized even if the dataset does not possess any distance-based cluster structure.

This work is structured as follows. After introducing the challenges and pitfalls of cluster analysis in five subsections, the results are divided into the following three subsections.

Two examples for the pitfalls of optimizing or selecting algorithms by the Davies–Bouldin index are presented directly in the result section. Other datasets and unsupervised indices are evaluated in SI C and SI E. In SI D: the MD plots reveal biases and the various states of probability outlining that benchmarking clustering algorithms with first-order statistics, box plots, or a small number of trials is not advisable. In addition, this is also shown in Fig. 2, left and SI C, Supplementary Fig. 10 left for high-dimensional datasets. The third subsection of the results outlines the first step for an unbiased benchmarking of clustering algorithms, for which the results of all evaluations based on appropriately used supervised indices are summarized in Table 1. This work finishes with the methods section explaining the selection process for datasets with clearly predefined structures allowing for only one correct partition of the data, the choice of high-dimensional datasets, evaluation criteria and access to state-of-the-art clustering algorithms.

**Table 1:** Typical distance-based clustering challenges with one example dataset each. The table summarizes the results of SI C, Supplementary Fig. 10 and SI D Supplementary Figs. 11–14. No algorithm is able to reproduce all types of problems with highly stable results. The challenge that no distance-based cluster structures exist is not included in this table because benchmarking is not possible in this case. Note that the benchmarking performed here does not allow the deduction if an algorithm fails due to the cluster structures or due to the distribution of the data.

Distance-based cluster structures	Exemplary dataset dimensionality d range of cluster size	Stable clustering solution	Small bias with minor variance	Small bias and unstable clustering solution (multimodality)	Large bias
Non-overlapping convex hulls with varying intra-cluster distance	Hepta, D = 3 14%-15%	24/41	QT, SOM,	CrossEntropyC, Hartigan, HCL, HDD, LBG, mvnpEM, npEM, Orclus, SOM Sparse k-means Spectral,	Diana, ProClus, RTC, PPC
Overlapping convex hulls	Atom D = 3 50%	10/41	DBS	CrossEntropyC	29/41
Non-overlapping convex hulls with varying geometric shapes and noise	Lsun3D D = 3 24–49% (Additionally, 4 outliers as noise)	Clustvarsel, , Gini, HDBSCAN, Minimax ModelBased, mvnpEM, npEM, VarSelLCM, Ward, , ,	Fanny, DBS, Orclus, CrossEntropyC, HDD	Spectral, ProClus	25/41
Linear non-separable entanglements	Chainlink D = 3 50%	DBS, Gini, HDBSCAN,mvnpEM, SingleL, Spectral, Spectrum, ,	Clustvarsel, CrossEntrpoy, Modelbased, npEM, VarSelLCM	/	29/41
High dimensionality with highly imbalanced cluster sizes	Leukaemia D = 7447 Range of cluster sizes: 2.7–50% (Additionally, 1 outlier as noise)	AverageL, CompleteL Diana, SingleL, WPGMA	DBS	Clara, HCL, QT	32/41 with Clustvarsel, CrossEntropy, ModelBased, mvnpEM, npEM, Orclus, RTC, and Spectrum not computable
High dimensionality with an unstable clustering solution	Cancer D = 18,167 Range of cluster sizes: 10%-17%	Gini	Ward	DBS, Hartigan, HDD, LBG, Neural Gas	34/41 with Clustvarsel, CrossentropyC, ModelBased, mvnpEM, npEM, Orclus, RobustTrimmedC, SparseH and Spectrum not computable

## Challenges and pitfalls

This work is based on two assumptions. First, there exists only one optimal partition of data defining the real clustering situation, which is contrary to the axioms of Kleinberg^13^. The existence of only one optimal partition will not hold for many clustering applications (c.f.^14^), but we assume that in the case of biomedical applications, it is a valid assumption for diagnoses or therapies (see description in SI A, Supplementary Figs. 1–4). Second, a trained physician or diagnostic specialist is able to recognize and validate the patterns of data for datasets in two or three dimensions (e.g.,^15^). Thus, empirically based clusters such as "diagnoses" should match the algorithmic clustering approach's results. If artificial datasets are defined systematically (e.g., in^16,17^), then manual clustering would be consistent with the prior classification.

Keeping these two assumptions in mind, in principle, five categories of challenges can be identified: data-specific cluster structures, the limitations of clustering criteria, the biases induced by evaluation, high-dimensionality and estimating the number of clusters.

### Challenge induced by clustering criteria

Clustering criteria make implicit assumptions about data^18–22^, resulting in biased clustering. Moreover, clustering algorithms partition the data even if the data do not possess distance-based structures^22,23^. No algorithm exists that is able to outperform all other algorithms if more than one type of problem exists^24^. More precisely, the insights of Geman et al.^25^ and Gigerenzer et al.^26^ state that the error in various types of algorithms is the sum of the variance, bias, and noise components, which is the starting hypothesis of this work. Here, the bias is the difference between the given cluster structures and the ability to reproduce these structures. If a global clustering criterion is given that an implicit definition of a cluster exists, the bias is the difference between this definition and the given structures in data. The variance is the stochastic property of not reproducing the same result in different trials. Small or zero variance means high reproducibility. Outliers in distance-based datasets can represent the data noise.

### Challenges in evaluating clustering solutions

Quality evaluation in unsupervised machine learning is often biased. This bias can be shown for quality assessments for clustering methods in the case of unknown class labels (unsupervised quality measures)^20^ as well as quality assessments for dimensionality reduction methods if graph theory insights are applied^23,27^.

In the case of supervised indices, partition comparison measures can yield the same results for a different clustering solution. Ball and Geyer-Schulz proved that all partition comparison measures they had found in the literature fail on symmetric graphs because they are not invariant w.r.t. the group automorphisms^28^. They state that given the automorphism group, their results on the decomposition of the measures generalize to arbitrary cluster problems^28^. Their analyzed supervised indices are available in the R package ‘partitionComparison’ CRAN (https://CRAN.R-project.org/package=partitionComparison). Since most of the real-world graphs contain symmetries^29^ and distance-based cluster structures can be described through graph theory^23^, the author agrees with^28^ that this insight is generalizable to clustering problems. Clearly, this theory developed by Ball and Geyer-Schulz means that different partitions of the data may result in the same value for a supervised quality measure (QM). In practice, this means that the usual definition of the F1 score has a probability to evaluate well-partitioned data and incorrectly partition data equally if enough algorithms and datasets are investigated. In conclusion, performing streamlined evaluations and comparisons of the clustering algorithms (e.g.^4^) can be inappropriate, especially as the number of trials and algorithms and parameters increases.

### Challenges for distance-based cluster structures

When a new method is proposed, quality assessment is performed with preselected supervised indices depending on the publication^30,31^. Either elementary artificial datasets are used without the precise investigation of the cluster structure (especially if they are distance- or density-based clusters) or natural datasets with unknown (or undiscussed) structures are selected. An evaluation is then performed using a priori, possibly arbitrarily given the classification, but it remains unknown if only one valid clustering scheme exists for these datasets. If more than one valid clustering scheme is possible, the discussion about algorithm performance becomes infeasible.

Such cases do not generally imply how well clustering algorithms work or indicate which structures an algorithm can find. More importantly, the reproducibility of a method is usually investigated insufficiently, meaning that methods can possess different states of probability depending on the trial, which remains invisible if first-order statistics or box plots are used.

### Challenges of high-dimensionality

Usually, three types of automatic approaches can be applied to cope better with high-dimensional data (c.f.^32–35^). In the first type, clustering is combined with dimensionality reduction (e.g.^36^), in the second type, clustering is combined with feature selection (c.f.^37^), and in the third type, deep learning is employed to learn feature representation for clustering tasks^38^.

Well-known approaches for the first type are subspace clustering (e.g.^39,40^) and clustering combined with various linear and non-linear projection methods. An extensive discussion of these methods can be found in^41^. Here, several algorithms of this type are selected (see SI F, Table 1).

For the second type, the best accessible approaches are based on finite mixture modeling which provide the framework for cluster analysis based on parsimonious Gaussian mixture models^42^. An extensive discussion of these methods can be found in^32^. Scrucca and Raftery proposed to use the Bayesian information criterion to compare mixture models fitted on a nested subset of features^42^. Alternatively, Marbac and Sedki proposed a new information criterion for selecting a model with relevant features^43^, which assumes that variables are independent within components^44^.

In a non-model-based clustering method, a lasso-type penalty to selected features was used in the so-called sparse clustering^45^. As another option, Random forests provide a proximity measure that can capture different levels of co-occurring relationships between variables and can be converted into an unsupervised learning method, for which the derived proximity measure can be combined with a clustering approach^46^. However, there is no proof that methods that integrate feature selection in clustering outperform a two-stage approach in which the first stage screens for relevant features and the second stage applies conventional clustering methods on the pre-selected features^47^. Nonetheless, Azizyan et al. conjecture that there could be certain conditions under which simultaneous feature selection and clustering would outperform the two-stage apporach ^47^. Furthermore, reducing the dimensionality of data automatically without taking into account the clustering goal can conduce to suboptimal results^32^.

In the third type of approach, the so-called deep clustering integrates representation learning and clustering as a single process to obtain the optimal representation space for clustering^48^. Karim et al^49^ claims that accuracy of non-deep learning clustering algorithms for high-dimensional datasets degrades drastically due to the curse of dimensionality (c.f.^32–35^). Such approaches mainly focus on image datasets, for example, see^50^, while few attempts have been made on documents^51^, for example, see^48^, and graph datasets (e.g.^52^). One disadvantage of such approaches is their lower robustness: small perturbations in the input space will lead to diverse clustering results since labels are absent in the unsupervised clustering task^48^. Some methods like structural deep clustering network^53^ can also be used on numerical data but require the computation of a specific graph based on the raw data as a input. In this case, clustering results will depend on the type of graph used because it defines the relevant neighborhoods in the data^23^. Recently deep kernel learning methods were proposed for clustering^54^. However, the corresponding infinite-dimensional minimization problem can be recast into a finite-dimensional minimization problem which can be tackled with non-linear optimization algorithms^55^. As a consequence of this Representer theorem, deep kernel learning clustering methods have similar optimization procedures to conventional algorithms like k-means with specific objective functions that may not be appropriate to the given structures in data. It should be noted that in the difference to state-of-the-art clustering methods (c.f. summary of over 60 algorithms and many clustering libraries in the R package ”FCPS” on CRAN), deep clustering algorithms were so far either directly inaccessible or only published as GitHub packages (see overview in^49^). In contrast, rigorous external testing and strict documentation guidelines are required for a submission of packages to the comprehensive R archive network (CRAN)^56^ which results in a high stability of algorithms and reproducibly of results independent of the system architecture. As a consequence, deep learning clustering methods with access restricted to GitHub often only work for specific system architectures, have unclear or inaccessible dependencies, and without clear documentation may yield incorrect results due to inappropriate usage.

In addition to these types of approaches, the challenge of high dimensionality for cluster analysis can be resolved via the selection of an appropriate distance metric (see^57^ for details).

### Estimating the number of clusters

Automatically determining the number of clusters has been one of the most difficult problems in data clustering^58^. Methods for automatically determining the number of clusters cast either into the problem of model selection^58^ or the number of clusters can be determined visually. Exemplary for the first case serve information criteria like AIC and BIC with which the number of Gaussian mixture components can be estimated (e.g.^59^). Further, Keribin demonstrated the consistency of BIC for selecting the number of components in mixture models ^60^. Clustering quality measures can be used which are typically based either on covariance matrices, or the intra, or intercluster distances can be compared to evaluate the homogeneity versus heterogeneity of the clusters (e.g.,^61,62^). For example, the distortion measure evaluates the average distance, per dimension, between each observation and its cluster center^63^. Further approaches use the gap statistic^64^, the quantization error^65^, the diversity index^66^ or by a novel approach of cross-validation for unsupervised learning^67^.

Visually, the most basic approach to estimate the number of clusters would be the elbow criterion^68^. Since then, various decision graphs or other visualization approaches for specific clustering methods were proposed^31,69^. Restricted to hierarchical clustering algorithms, large changes in fusion levels of the ultrametric portion of the used distance measure indicate the best cut, and, hence, the number of clusters^23^. Furthermore, scatter plots of dimensionality reduction methods (so-called projection methods^70^) , more elaborate methods based on emergent self-organizing maps^71^, or even interactive methods from the field of visual analytics (e.g.^72^) can be used. At last, some clustering algorithms automatically select the number of clusters (e.g.,^30,73–75^). Since this wide variety of possible approaches for estimating the appropriate number of clusters, the evaluation of this critical parameter will not be performed in this study. If the parameter has to be set, it will be set by a predefined number of clusters.

## Results

The results are divided into three parts. In the first two sections, the reasons that clustering is biased and cannot be optimized without prior knowledge are shown. The third section outlines the first step in an unbiased benchmarking of clustering algorithms.

### Optimizing parameters based on an unsupervised QM imposes bias

**Figure 1:**
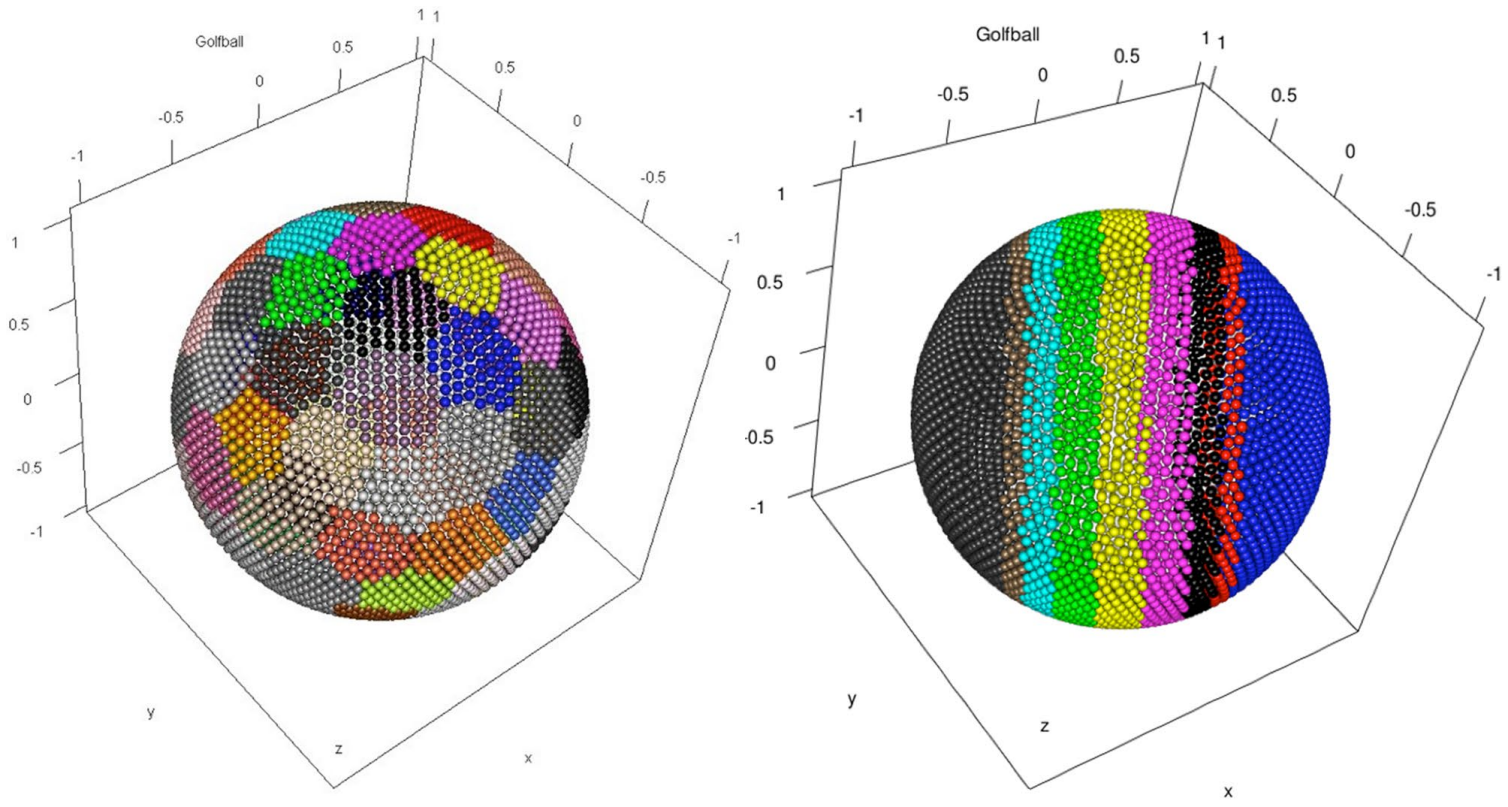
The coloured points of the two SOM clusters of the GolfBall dataset^16^. The figure on the left shows an optimal clustering of 0.83 for the Davies–Bouldin index, and the figure on the right shows the worst case of 11.8 for the Davies–Bouldin index.

**Figure 2:**
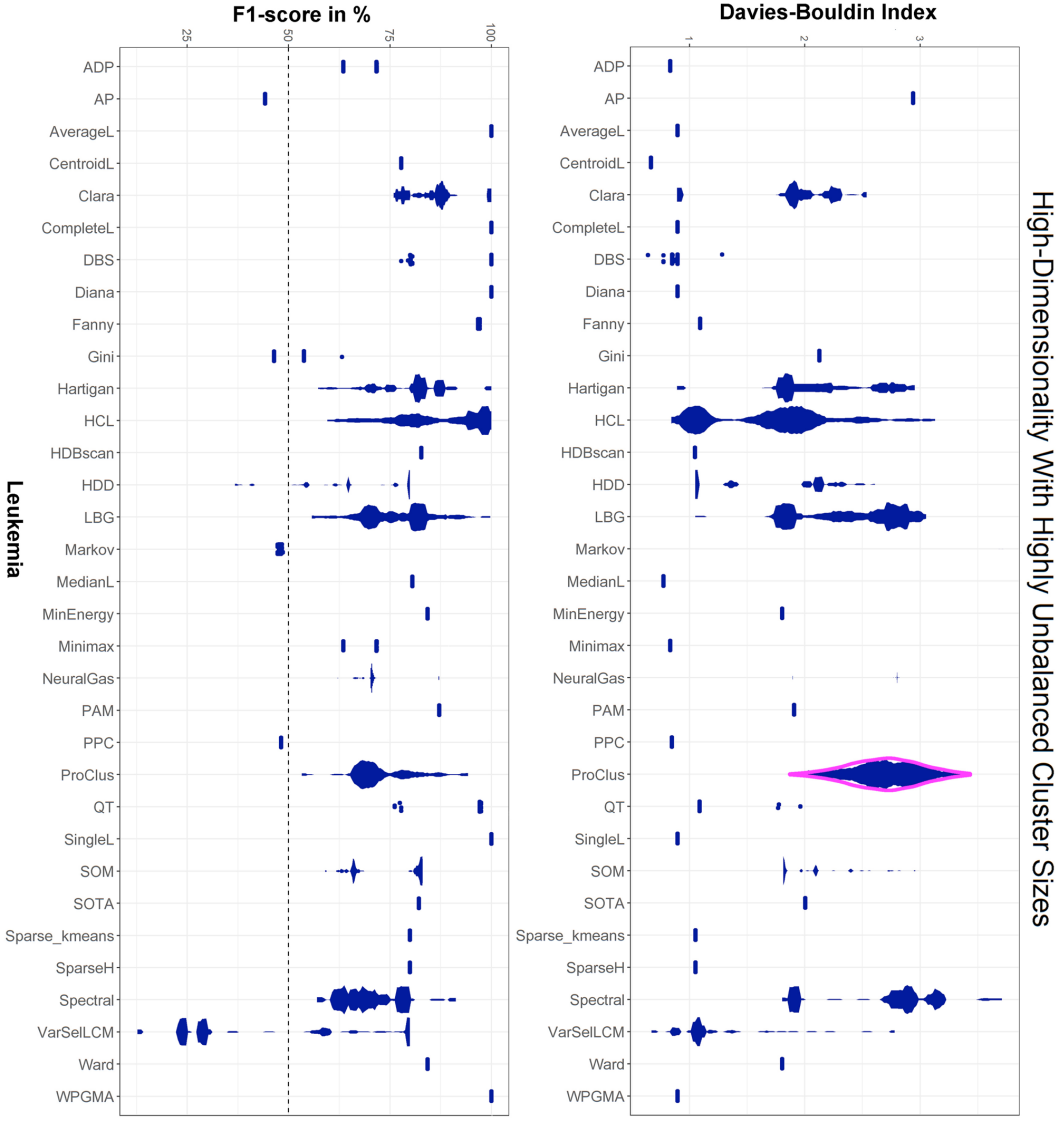
MD-plots of the micro-averaged F1 score (left) and Davies–Bouldin index (right) across 120 trials for 33 clustering algorithms calculated on the leukaemia dataset. Distance-based structures with imbalanced classes are not easy to tackle in high-dimensional data. The chance level is shown by the dotted line at 50%. The choice of an algorithm by the Davies–Bouldin index would lead to the selection of the CentroidL or for some trials VarSelLCM algorithms, whereas using the ground truth shows that AverageL, CompleteL, DBS, Diana SingleL and WPGMA are appropriate algorithms to reproduce the high-dimensional structures with low variance and bias. The results for Clustvarsel CrossEntropyC, ModelBased, mvnpEM, npEM, Orclus, RTC, and Spectrum could not be computed. Note that, Markov clustering results in only one cluster in which case the Davies-Bouldin index is not defined.

If no distance-based cluster structures exist, most algorithms will still partition the data^22,23^, and unsupervised evaluation criteria will provide valid values. Two clustering solutions are provided for which a clustering algorithm yields a homogenous grouping for data without distance-based structures (Fig. 1). Optimization of 9 parameters of SOM clustering can vary in Davies–Bouldin indices^10^ between 11.8 and 0.83 (Fig. 2). However, for both cases, the class-wise inter-cluster distance distribution remains with a variance equal to that of the full distance distribution (SI B, Supplementary Figs. 5 and 6).

### Using an unsupervised QM for the chosen algorithm imposes bias

The evaluation of 24 conventional clustering algorithms is presented with the mirrored density plot (MD-plot)^76^ in Fig. 2.

The MD-plot of the micro-averaged F1 score in Fig. 2 (right) visualizes the estimated probability density functions (PDF) for each clustering algorithm across 120 trials. First, multimodality for the DBS, Clara, Hartigan, LBG, ProClus, SOM, spectral, HCL, and QT clustering algorithms is clearly visible. Using the ground truth, the PDFs of supervised quality measure (QM) for each algorithm show that AverageL, CompleteL, DBS, Diana SingleL and WPGMA are appropriate algorithms to reproduce the high-dimensional structures. However, the stochastic nature of DBS, Clara, and HCL yields different states of the probability of which only one state is appropriate. The MD-plot of the Davies–Bouldin index suggests the Markov, MinEnergy, and HCL are appropriate algorithms. An additional high-dimensional example with a balanced number of instances also leads to inappropriate algorithm selection (SI C Supplementary Fig. 10), although approaches of knowledge discovery indicate a distance-based cluster structure (SI A, Supplementary Figs. 3 and 4).

### Benchmarking shows bias and multimodal variance

Table 1 shows the summarized results of the MD-plots in SI C and D (Supplementary Figs. 10–14). Here, the error rate is chosen because the number of instances per class is not highly imbalanced except for outliers, which are defined as noise. Table 1 validates the claim of^77^ because it shows that each global clustering criterion imposes a particular structure on the data, and only if the data happen to conform to the requirements of a particular criterion are the actual clusters recovered.

## Discussion

The bias and reproducibility of specific distance-based cluster structures were investigated systematically using 41 clustering algorithms. The results show the pitfalls of

1. Parameter optimization on datasets without distance-based cluster structures.
2. Algorithm selection by unsupervised quality measures on biomedical data.
3. Benchmarking clustering algorithms with first-order statistics or box plots or a small number of trials.

The clustering performance on two biomedical datasets (Fig. 2 and SI C. Supplementary Fig. 10) indicates that the evaluation of datasets and algorithms with the Davies–Bouldin index, the Dunn index (SI E, Supplementary Fig. 16), and the average silhouette value (SI E, Supplementary Fig. 15) does not enable researchers to select an appropriate clustering algorithm or result that is contrary to prior claims^4^. The best-performing algorithms are often inappropriate for these datasets since prior knowledge about high-dimensional data (presented in SI A, Supplementary Figs. 1–4) reveals that bias is induced by evaluating the Davies–Bouldin index, the Dunn index (SI E, Supplementary Fig. 16) or the average silhouette value (SI E, Supplementary Fig. 15). Recent research reports a significant correlation between the F1 score and silhouette values^4^. However, a correlation does not necessarily mean that a valid relationship between two quality measures (or any two variables) exists (see the counter-example in^78^). Here, the silhouette value is misleading for every clustering algorithm because it investigates whether the cluster structures are spherical^23^.

This is a general problem of algorithm selection by unsupervised quality measures because such an approach solely evaluates how well a clustering algorithm is able to partition the data into structures with a specific assumption about the data. Unsupervised quality measures possess biases^20^ requiring specific assumptions about the data. Furthermore, instead of selecting algorithms by an unsupervised quality measure, one could also optimize the unsupervised quality measure directly by defining an objective function based on this unsupervised index which would achieve the same goal.

Typically, there are two alternatives for selecting algorithms with unsupervised quality measures. First, assumptions about the structures in data can be investigated manually with knowledge discovery approaches before algorithm selection (e.g., see SI A) or be based on other sources of prior knowledge. An example of prior knowledge could be the assumption that clusters should possess interrelations to external medical factors like survival.

As a consequence of investigating the structures in data before cluster analysis, natural high-dimensional datasets are only valuable for benchmark algorithms if the structures are known beforehand and the prior classification is unambiguous, which is often not the case or remains undiscussed. Otherwise, benchmarking with high-dimensional datasets and unsupervised quality measures is biased and could be misleading.

Secondly, the author follows the argument of Holzinger^79^ that the integration of a Human-in-the-loop’s (HIL)'s knowledge, intuition, and experience can sometimes be indispensable, and a HIL's interaction with the data and algorithm selection and optimization can significantly improve the overall ML pipeline. Such interactive ML uses the HIL to make possible what neither a human nor a computer could do alone (cf.^79^). The HIL is an agent that interacts with algorithms, allowing the algorithms to optimize their learning behavior (cf.^80^). This perspective fundamentally integrates humans into the algorithmic loop with the goal of opportunistically and repeatedly using human knowledge and skills to improve the quality of clustering algorithms (cf.^80^, see also^81,82^). For example, interactive projection-based clustering integrates the HIL in the process of algorithm and parameter selection^72^.The first results section serves as an example of the pitfall in cluster analysis that if no distance-based structures exist, then algorithm selection and parameter optimization by evaluating an unsupervised quality measure will not lead to any meaningful results. For high-dimensional data, the existence of cluster structures has to be investigated prior to using such a dataset for benchmarking. Both results imply that optimizing parameters and selecting algorithms without prior knowledge about the data results in an implicit restriction of the cluster structures that are sought even if they do not exist. This work outlines that optimization contradicts the typical knowledge discovery approach for biomedical data. If the values of any unsupervised quality measure are optimized, implicitly, a new clustering algorithm is created that possesses this quality measure as its global criterion. Without extensive prior knowledge, either based on medical insights or various knowledge discovery approaches, automation in cluster analysis for knowledge discovery can be inappropriate.

In the third part, 41 clustering algorithms are compared on artificially defined data structures and well investigated high-dimensional data with specifically defined distance-based challenges, revealing the biases of these clustering algorithms. Evaluating 120 trials per algorithm enables the visualization of the PDF of each algorithm’s error rates (SI C and SI D, Supplementary Figs. 10–14). The benchmarking uncovers variance in half of the algorithms investigated (SI F Table 1) and multimodalities in the variances of F1 score and error rate in these algorithms, meaning that these algorithms have different states of probabilities, and for noisy datasets, sometimes no stable clustering solution can be generated. This finding means that first-order statistics such as the mean and standard deviation or even box plots are invalid to compare the results of quality measures.

The resulting clusterings of algorithms typically have either a large variance and a small bias (e.g., spectral clustering and DBS clustering) or a large bias w.r.t. the distance-based structures investigated and a small variance in the results (e.g., hierarchical clustering algorithms). The exceptions are the two k-means clustering algorithms, which have high variance and high bias. Surprisingly, subspace clustering algorithms are unable to deal with the overlapping convex hulls of Atom, in which the low-dimensional manifold would be one-dimensional, and the high-dimensional datasets. It seems that subspace clustering is inappropriate for distance-based datasets. Spectral clustering is clearly affected by noise, and model-based clustering cannot be used if the dimensionality increases significantly. DBS is the only algorithm that exploits emergence, which results in the ability to reproduce every structure type because no global clustering criterion is required. However, it possesses a considerable variance that often has to be extensively dealt with. The claim of Karim et al.^49^ that the accuracy of non-deep learning clustering algorithms for high-dimensional datasets degrades drastically due to the curse of dimensionality was disproved for up to D > 18.000 with the exception of model-based clustering algorithms. There are sufficient many state-of-the-art methods available that can cope with high-dimensionality. Although a high number of cases (N > 10^5) was not investigated in this work, based on the evaluated results, it can be assumed that several non deep learning clustering algorithms will compute results with comparable efficiency.

In sum, the authors do not want to make any recommendation of which clustering algorithm outperforms the others, because a complete benchmarking study should be double-blinded and unbiased, meaning that the authors of the study should not be the inventor of one of the methods, the authors should not know themselves which algorithm is which before ending the study and the reviewers should not know which authors performed the study.

However, two points are evident. First, the results of clustering algorithms should be compared over many trials on previously extensively investigated datasets with various knowledge discovery approaches (e.g., SI A, Supplementary Figs. 1–4) or precisely defined artificial datasets with a specific pitfall. Second, global clustering criteria and unsupervised and supervised quality measures in cluster analysis possess biases and can impose cluster structures on data. Only if the data happen to meet the structure type is appropriate validation of the clustering solution possible. Therefore, either various knowledge discovery approaches or Human-in-the loops (HILs) are necessary before a highly automated approach for cluster analysis such as ClustEval^9^ is applied.

On the one hand, the results shown here are not generalizable in the sense that an algorithm reproducing the distance-based structure is always able to reproduce the structures of this type. On the other hand, the results show the clustering algorithms' limitations if distance-based data structures are investigated. Suppose an algorithm is not able to reproduce structures of a particular type in any trial. In this case, it is fairly improbable that that algorithm will be able to reproduce such types of distance-based structures in high-dimensional data or that extensive parametrization will significantly reduce the bias.

## Conclusion

This work emphasizes that only the combination of empirical medical knowledge and an unbiased, structure-based choice of the optimal cluster analysis method w.r.t. the data will result in precise and reproducible clustering with the potential for knowledge discovery of high clinical value. It reveals the challenges of benchmarking and automation of cluster analysis for knowledge discovery. Unbiased benchmarking of clustering should be performed using artificial or extensively investigated datasets to compare the clustering results with clearly defined cluster structures. Then, combining the Mirrored Density plot (MD-plot) with a supervised quality measure of apparent and exploitable bias is a possible solution to evaluate clustering algorithms. The bias in the quality measure has to depend on the dataset.

It is open to the reader to interpret the results and favor some algorithms because it is visible that on average, two out of three conventional clustering algorithms fail even on the most straightforward datasets if structures based on the relationships between data points are of interest. Future research should systematically investigate if deep learning clustering can reproduce structures in data better than state-of-the-art algorithms, although based on current literature review it is questionable.

## Methods

Benchmarking will be performed on two high-dimensional datasets (D > 7000 and D > 18,000) and four artificially defined data structures with 41 clustering algorithms that are available in a previously published clustering suite^83^. The high-dimensional datasets possess one true partition of the data, which was verified by various methods and a domain expert.

### Generation of distance-based structure in data

The following different distance-based challenges are provided for the task of separating data into homogeneous groups that are heterogeneous to each other. They defined distance-based cluster structures because all class-wise inter-cluster distances are larger than the full distance distribution (SI B, Supplementary Figs. 7–9, for detailed discussion please see^57^). If the dimensionality does not become too high, the full distance distribution is usually multimodal, which can be statistically tested^84,85^. These structures in data can be generated for arbitrary sample sizes and are described in one of the following ways:

- Linear separable clusters of non-overlapping convex hulls in which the intra-cluster distances can vary
- Cluster structures with overlapping convex hulls
- Cluster structures of varying geometric shapes and noise
- Complex entangled clusters that can be separated only non-linearly
- No existing distance-based structures

Five samples of these artificially defined structures in data are used^16^. They have a prior classification on clearly predefined structures allowing for only one correct partition of the data. The five artificially defined data structure types are called Hepta, Atom, Lsun3D, Chainlink, and GolfBall and are selected to address the issues mentioned above. Detailed descriptions can be found in^16^.

### Choice of high-dimensional datasets

Additionally, for cluster analysis, the issue of high dimensionality can arise, which is often coupled with various effects such as the curse of dimensionality in which distance measures become meaningless. Moreover, cluster structures with a highly imbalanced number of instances per cluster can be of interest. Thus, we select two high-dimensional datasets (d = 7700 and d = 18,000) with distance-based structures (SI A, Supplementary Figs. 1–4).

The first one, the leukaemia dataset (see SI A for a description) with medical diagnoses provided by experts was selected because it has a high dimensionality by measuring more than 7000 gene expression levels simultaneously, the cluster sizes are highly imbalanced and acute myeloid leukaemia (AML) and chronic lymphocytic leukaemia have been accepted as clearly separable entities for many centuries. Only 8 cluster diagnoses have been proposed for AML^86^, and these categories have only recently been expanded with respect to specific molecular events^87^. Moreover, AML is a cancer in which the number of driver mutations in genes required during oncogenesis is relatively small^88^.

SI A outlines that a clear sub-manifold can be detected in which the subtypes of leukaemia (i.e., CLL, AML, APL (formerly M3 leukaemia according to the Bennett FAB classification)) are clearly separable from each other and from healthy individuals. Supplementary Figs. 1 and 2 outline a clear distance-based cluster structure of the prior classification of an imbalanced number of cluster instances. The dataset possesses an unambiguous ground truth because it reflects disease entities with entirely different therapy approaches. CLL is treated differently from AML, and the AML subgroup APL is treated differently from all other AML patients^89^, while healthy individuals do not require treatment at all. Despite the molecular diversity that leads to the previous AML categorization^87^, the dataset used here describes the most fundamental information, which is treatment modality.

In sum, the leukaemia dataset can be evaluated for the purpose of benchmarking, but the usual error rate would be unfavourably biased because the small clusters are highly relevant from a medical point of view. Thus, a specific F1 score has to be chosen as discussed in SI A because the error rate will have the inappropriate bias when weighting the small but relevant class of APL considerably lower than the large classes of AML and CLL.

The cancer dataset (see SI A for a description) possesses five types of diagnoses with more than 18,000 RNA-Seq gene expression levels. The class sizes are approximately balanced. Despite the high dimensionality of the dataset, distance-based cluster structures are still detectable (SI A, Supplementary Figs. 3 and 4). However, the dataset is noisier than the leukaemia dataset, and it can be expected that the given classification is an unstable solution w.r.t. the clustering of distance-based structures.

### State-of-the-art clustering algorithms and evaluation criteria

As suggested in^4^, for biomedical data, the comparison of an internal index is performed with the F1 score based on the ground truth, and the error rate is chosen. The error rate (1-accuracy) is used for which the quality measure's bias is clearly known. The accuracy measure does not penalize an incorrect clustering of a small class. Therefore, the artificially defined data structures used have equal-sized clusters except for one, in which the outliers are used to generate a tiny amount of noise in the data but are irrelevant w.r.t. the three main clusters.

For every cluster algorithm, the clustering accuracy is calculated across 120 trials. For each trial, the best of all the permutations of labels with the highest accuracy is selected because algorithms define the labels with arbitrary clustering w.r.t to the prior classification. Moreover, this approach avoids the problem described in^28^ because all permutations are investigated.

For highly imbalanced classes in the leukaemia dataset, the accuracy (error rate) has an inappropriate bias. Therefore, the micro-averaged F1 score^90^ cites^91^ is used as suggested by^92^. In this case, the best permutation is chosen, too, as described above.

All clustering algorithms and access to the artificial data structures are available in the 'FCPS' package^83^. The main parameter set is the number of clusters (NOC in Supplementary Table 1, SI F) if required by a method. In this work it is assumed that NOC is known prior to the clustering. Hence, the correct NOC for each dataset is used. If a kernel radius is required but no default value is accessible, it is estimated by the suggestion in^93^. If an additional parameter that has only two options is available, the best option regarding the accuracy is chosen. More advanced or numerical parameters are set to the defaults (SI F Supplementary Table 1). SI F provides a detailed overview of clustering algorithms and the abbreviations used in this work.

Given a valid quality measure for clustering, there are several approaches to evaluate the probability of a result if many trials are investigated (e.g.,^94^ and^95^).

In this work, a mirrored density plot (MD-plots), which is a schematic plot that visualizes the estimated PDF of "box-plot-like" features as violins^76^, is used. It was shown that the MD-plot outperforms comparable violin plots^76^ because its internal density estimation is particularly suitable for the discovery of structures in features, allowing the discovery of mixtures of Gaussians^96^. The MD-plot is available in the "DataVisualizations" R package from CRAN^76^ or in Python in the md-plot package on PyPI.

Computations were performed in either R 3.6.1 on Microsoft Azure using the VM size F64s_v2 with the specification of 64 CPUs, 128 GB RAM, 32 data disks, 80,000 max IOPS or an iMac Pro (late 2017), 2,3 GHz 18-Core Intel Yeon W, 256 GB RAM with the R package 'parallel' in R-core for parallel computation.

### Ethics approval

According to the Declaration of Helsinki, written patient consent was obtained for the leukemia dataset, and the Marburg local ethics committee approved retrospective calculation studies with this dataset (No. 138/16).

## Supplementary Information

Supplementary Information.

## Data Availability

The cancer dataset is available from the UCI ML repository (https://archive.ics.uci.edu/ml/datasets/gene+expression+cancer+RNA-Seq); other used data are published and accessible via^16^.

## Code availability

All algorithms are accessible in R or Python, as described in^83^.
