# Publisher Correction: Distance-based clustering challenges for unbiased benchmarking studies

**DOI:** 10.1038/s41598-021-99687-x

**Published:** 2021-10-06

**Authors:** Michael C. Thrun

**Affiliations:** 1 DataBionics AG, Mathematics and Computer Science, The University of Marburg, Hans‑Meerwein Str, 35032 Marburg, Germany; 2 Department of Hematology, Oncology and Immunology, Philipps-Universität Marburg, Hans‑Meerwein‑Straße 6, 04A28, 35032 Marburg, Germany

Correction to: *Scientific Reports* 10.1038/s41598-021-98126-1, published online 23 September 2021

The original version of this Article contained an error where the work of Keribin (2000) was incorrectly cited in the Challenges and pitfalls section under the subheading ‘Estimating the number of cluster’. As a result, Reference 60 was omitted from the Reference list.

“Further, Kerbin demonstrated the consistency of BIC for selecting the number of components in mixture models [Keribin, C.: Consistent estimation of the order of mixture models, Sankhyā: The Indian Journal of Statistics, Series A, Vol. 62(1), pp. 49-66, 2000].”

now reads:

“Further, Keribin demonstrated the consistency of BIC for selecting the number of components in mixture models^60^.”

The original Article has been corrected.

