# Supplementary material for: Distance-based clustering challenges for unbiased benchmarking studies
Source: Sci Rep. 2021 Sep 23;11:18988. doi: 10.1038/s41598-021-98126-1 (PMC8460803; doi:10.1038/s41598-021-98126-1)
Supplement: Supplementary file 1 — Supplementary Information. [file 41598_2021_98126_MOESM1_ESM.docx]

# **Supplementary Information**

## ***SI A: Discovering High-Dimensional Distance-Based Structures***

## Two examples provided below show that with the correct preprocessing approach and an appropriate choice of the distance measure, one can find a metric space where the relative relationship between high-dimensional data points becomes of interest to the domain expert. Then, the intra-cluster distances between high-dimensional points should have rather small values and a smaller variance and inter-cluster distances with larger values and a smaller variance (e.g., SI A, Supplementary Figs. 1 and 3) than those of the full distance distributions^1^.

## In the first example, the leukaemia dataset consists of 12,692 gene expression measurements from 554 subjects, as reported previously^2^. Each gene expression is a logarithmic luminance intensity (presence call), which was measured in primary bone marrow samples of 455 leukaemia patients and 105 healthy donor samples using Affymetrix technology as described in^2^. Fifteen patients were diagnosed with acute promyelocytic leukaemia (APL), 266 had chronic lymphocytic leukaemia (CLL), and 164 had acute myeloid leukaemia (AML). From a diagnostic point of view, AML and CLL are distinct disease entities, and APL is a subgroup of AML. Regarding therapy, all four groups received different treatments. The AML patients received combined cytarabine in a combined chemotherapy regimen, the CLL patients were treated with specific targeted therapies such as tyrosine kinase inhibitors (TKIs) or immune chemotherapy, the APL patients had an excellent prognosis with retinoic acid and arsenic trioxide treatment^3^, and the healthy individuals did not require treatment. The dataset was preprocessed. The distinctions are clearly visible in the small class-wise intra-cluster distances (Supplementary A*,* Fig. 1, left) and large class-wise inter-cluster distances (Supplementary A*,* Fig. 1, right) and the multimodal distance distribution (for Def. please see ^1^) of the whole dataset. The topographic map of the NeRV projection^4^ shows high-dimensional structures with four distinct valleys, one per class (Supplementary *A,* Fig. 2), reproducing the results computed by the ESOM projection^5^ and DBS projection^6^.


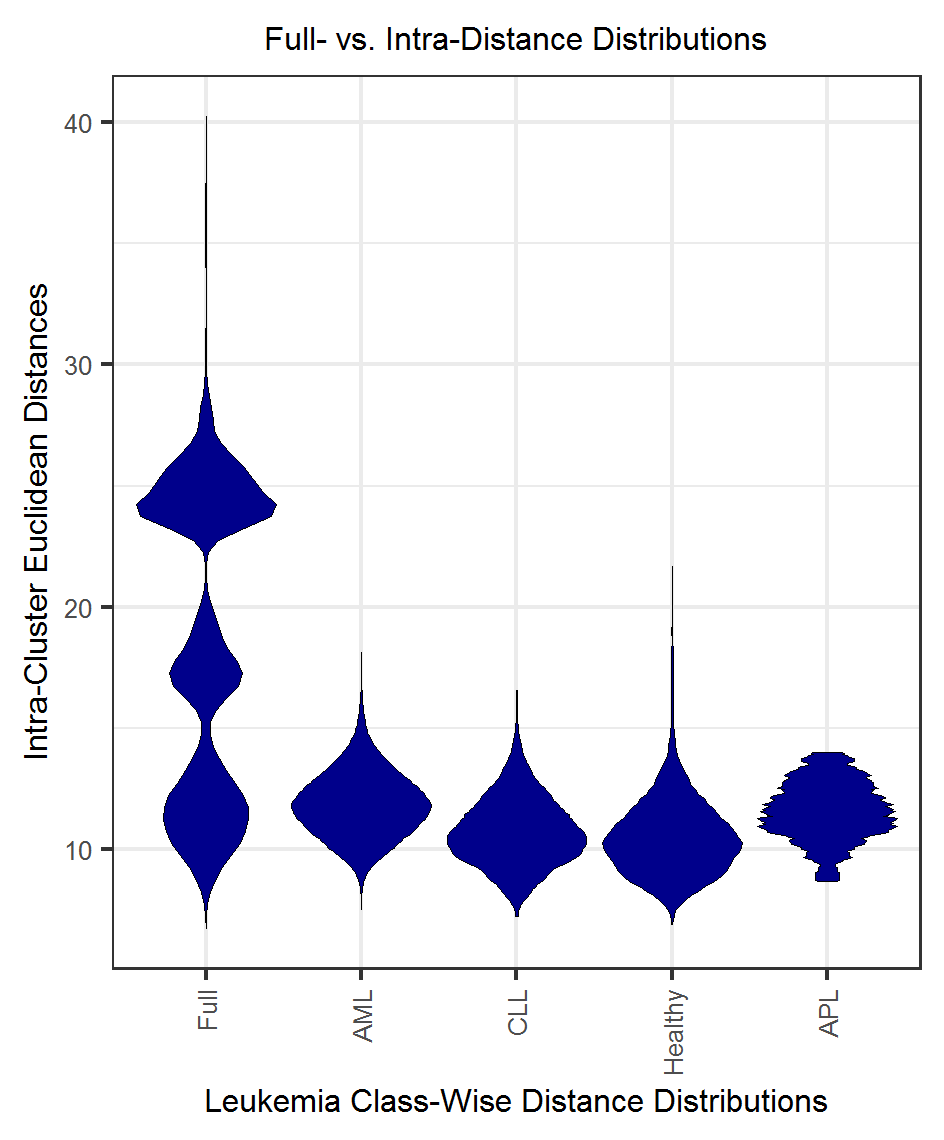

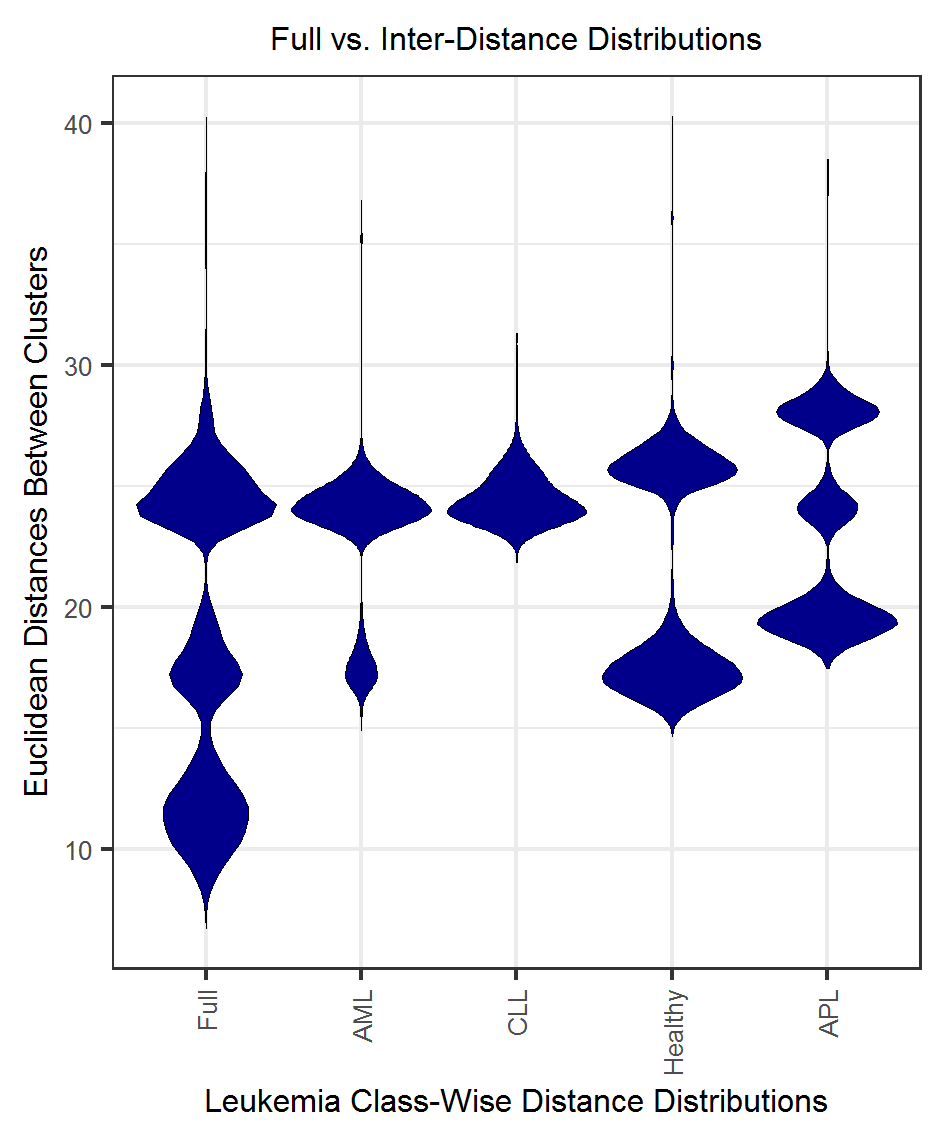


**Supplementary** **Fig. 1**. Inter- and intra-distance distributions from the leukaemia dataset reveal a clear distance-based structure because the distances in the clusters (left) are in the first mode of small distances of the full distance distribution and the distances between the clusters (right) are in the second and third modes of the full distance distribution (vast distances).


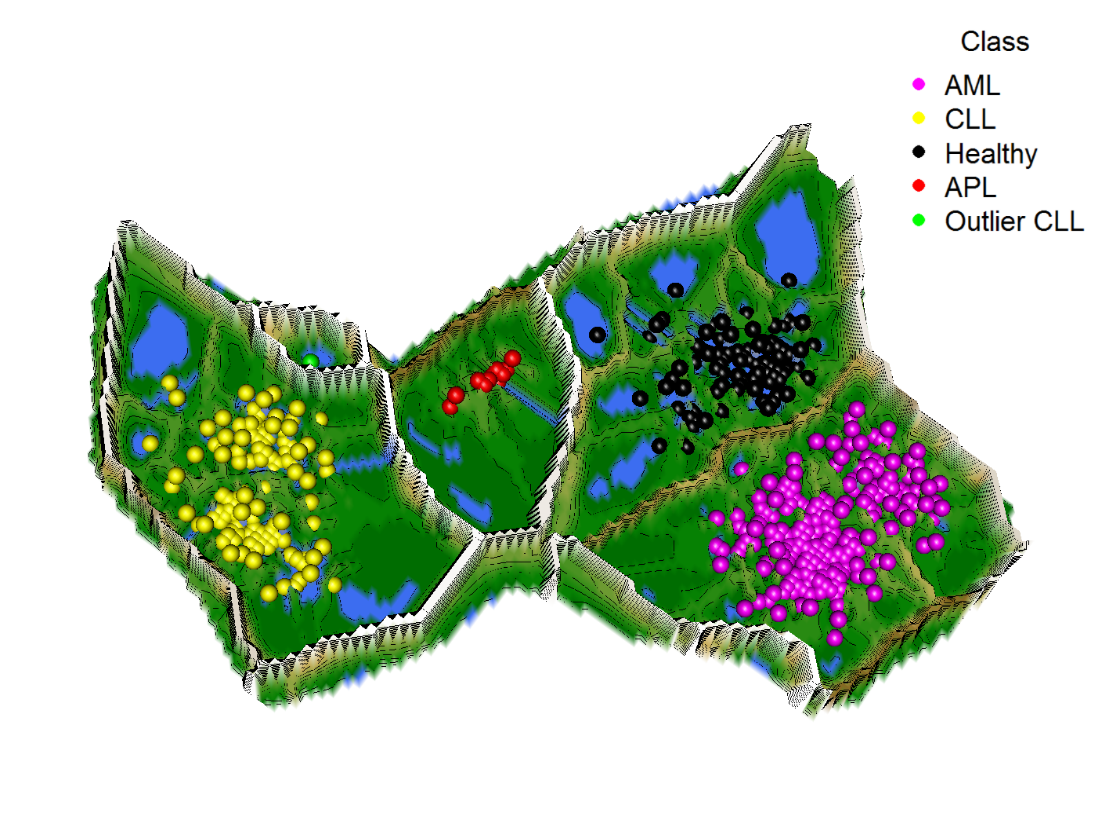


**Supplementary** **Fig. 2**. Topographic map of the high-dimensional structures of the leukaemia dataset using the NeRV projection^4^ and the generalized U-matrix^5^ reveals four valleys and one outlier in a volcano. Each subject is symbolized by one point. The projected points are coloured by the class labels defining the various illnesses as well as the healthy subjects. As valleys and classifications overlap, the topographic map indicates a clear cluster structure. Topographic maps also have the potential to reveal when a dataset does not possess any cluster structure at all^6-8^. The topographic map was generated with the R package “Generalized U-Matrix” available on CRAN^8^ (https://CRAN.R-project.org/package=GeneralizedUmatrix).

In the second example, the cancer dataset consists of 801 samples with 18,617 features, with each feature being the RNA-Seq gene expression level measured by the Illumina HiSeq platform. It consists of a sample of the data published in^9^ as a part of the Cancer Genome Project (c.f. https://www.sanger.ac.uk/science/groups/cancer-genome-project). The data are available from the UCI ML repository^10^ in which five types of diagnoses are given: lung adenocarcinoma (LUAD), breast carcinoma (BRCA), kidney renal clear-cell carcinoma (KIRC), colon adenocarcinoma (COAD), and prostate adenocarcinoma (PRAD).

Recent research indicates that the organ of origin is less relevant than the type of mutation, which could be the reason that the distance-based cluster structures are less distinct in Supplementary Figs. 3 and 4. Supplementary Fig. 3 indicates that, except for KIRC and LUAD, the class-wise distributions of intra-cluster distances are rather large compared to those of the full distance distribution. The inter-cluster distances in Supplementary Fig. 3 (right) are rather small compared to those of the full distance distribution. In the topographic map in Supplementary Fig. 4, five valleys are still visible, but many outliers and some misclassified points can be identified. Of note, the proximity of the two slightly overlapping valleys for PRAD and BRCA may indicate that the topographic map of the generalized U-matrix may be particularly useful for the definition of therapeutic subgroups because anti-hormonal treatment is the predominant treatment modality for these two cancers. In contrast, LUAD, KIRC, and COAD are treated with different chemotherapy regimens. In sum, the cancer dataset is noisier than the leukaemia dataset, and it can be expected that the given classification is an unstable solution w.r.t. the clustering of distance-based structures.


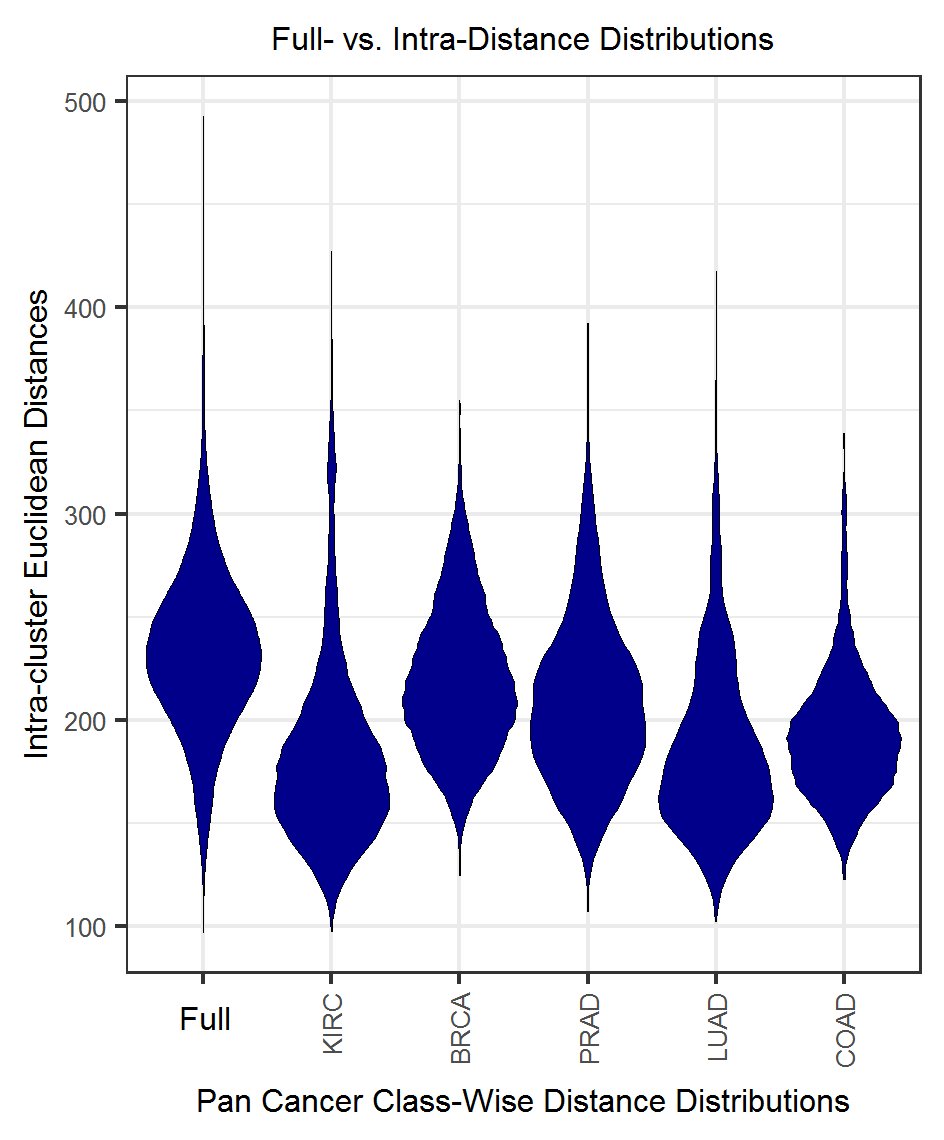

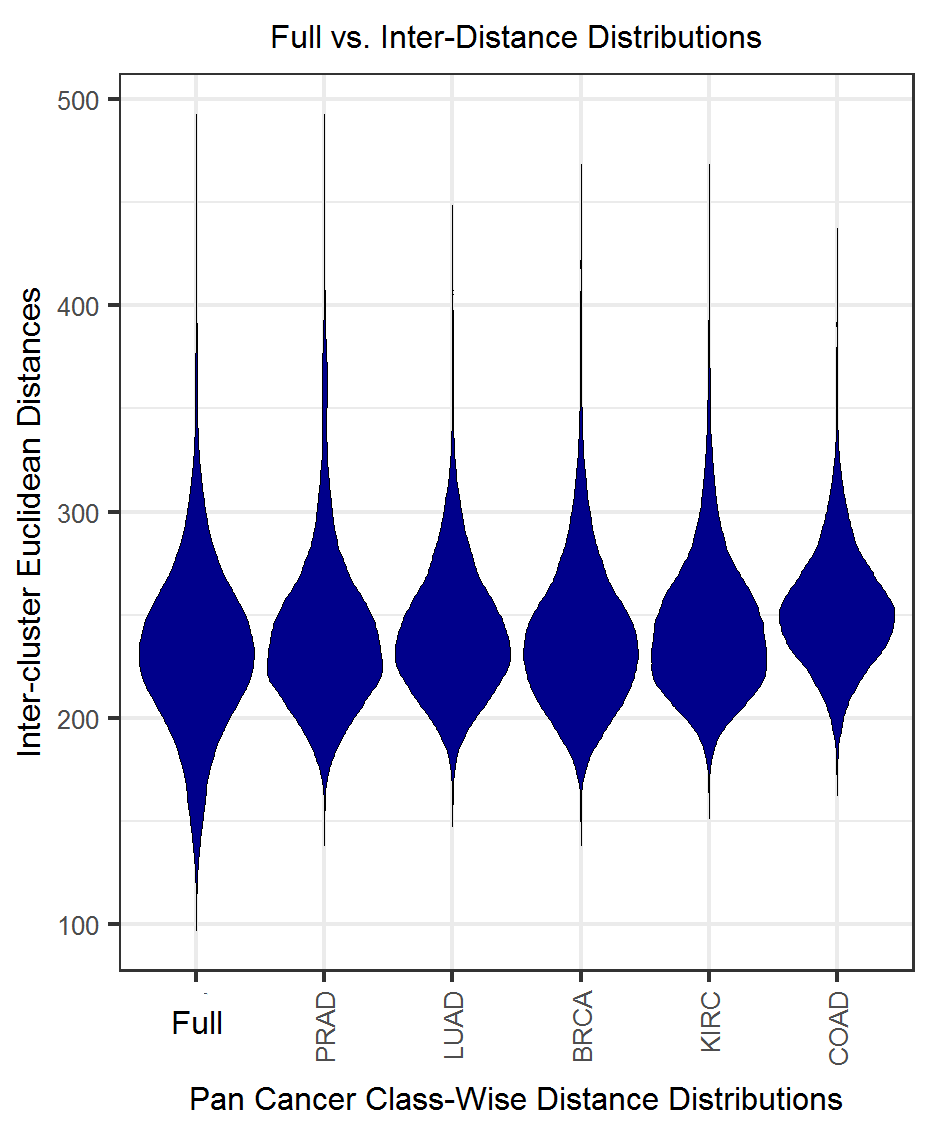


**Supplementary** **Fig. 3**. The inter- and intra-distance distributions form the cancer dataset reveal a clear distance-based structure because the distances in the clusters (left) are in the first mode of small distances of the full distance distribution and the distances between the clusters (right) are in the second and third modes of the full distance distribution (large distances).


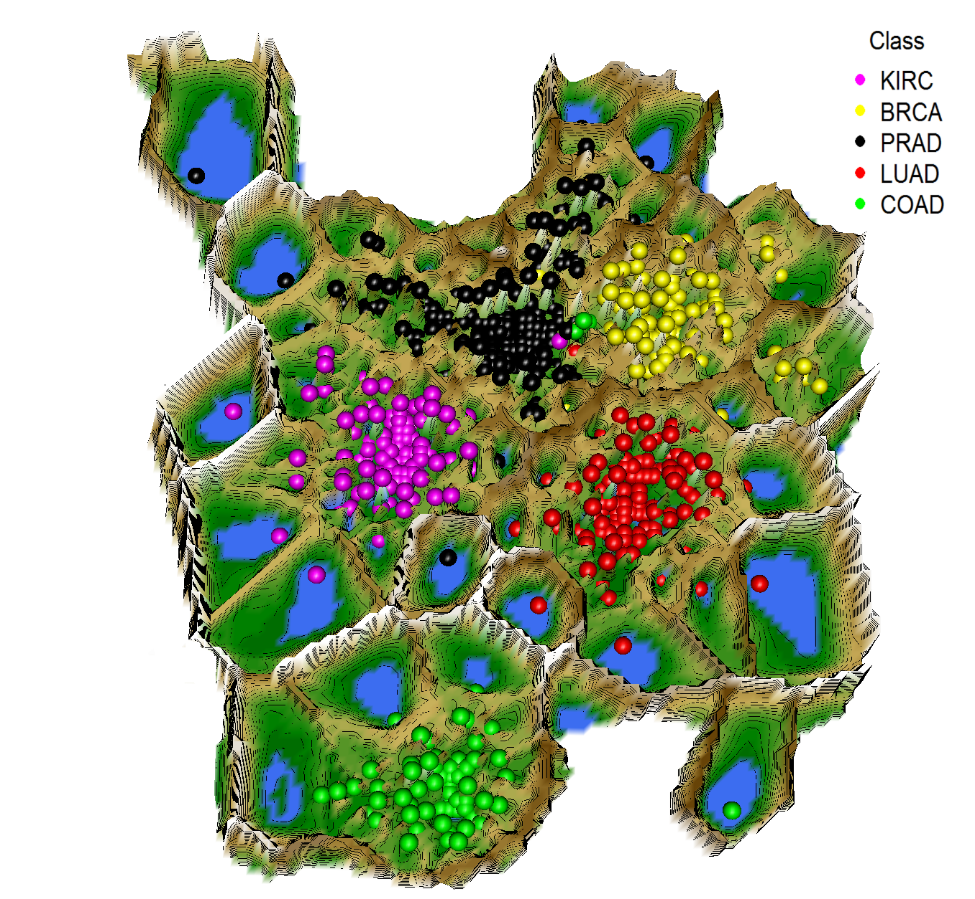


**Supplementary** **Fig. 4**. Topographic map of high-dimensional structures using the NeRV projection^4^ and the generalized U-matrix^5^ reveals five valleys and several outliers in volcanoes. Each subject is symbolized by one point with the diagnosis defined in the legend. The colours of the projected points indicate the class labels of the diagnosis. The topographic map indicates a cluster structure because the valleys and classification overlap. However, it seems that, especially between PRAD and BRCA, there are some misclassified points. The topographic map was generated with the R package “Generalized U-Matrix” available on CRAN^8^ (https://CRAN.R-project.org/package=GeneralizedUmatrix).

***SI B: Distance Distributions of Artificially Generated Data Structures***


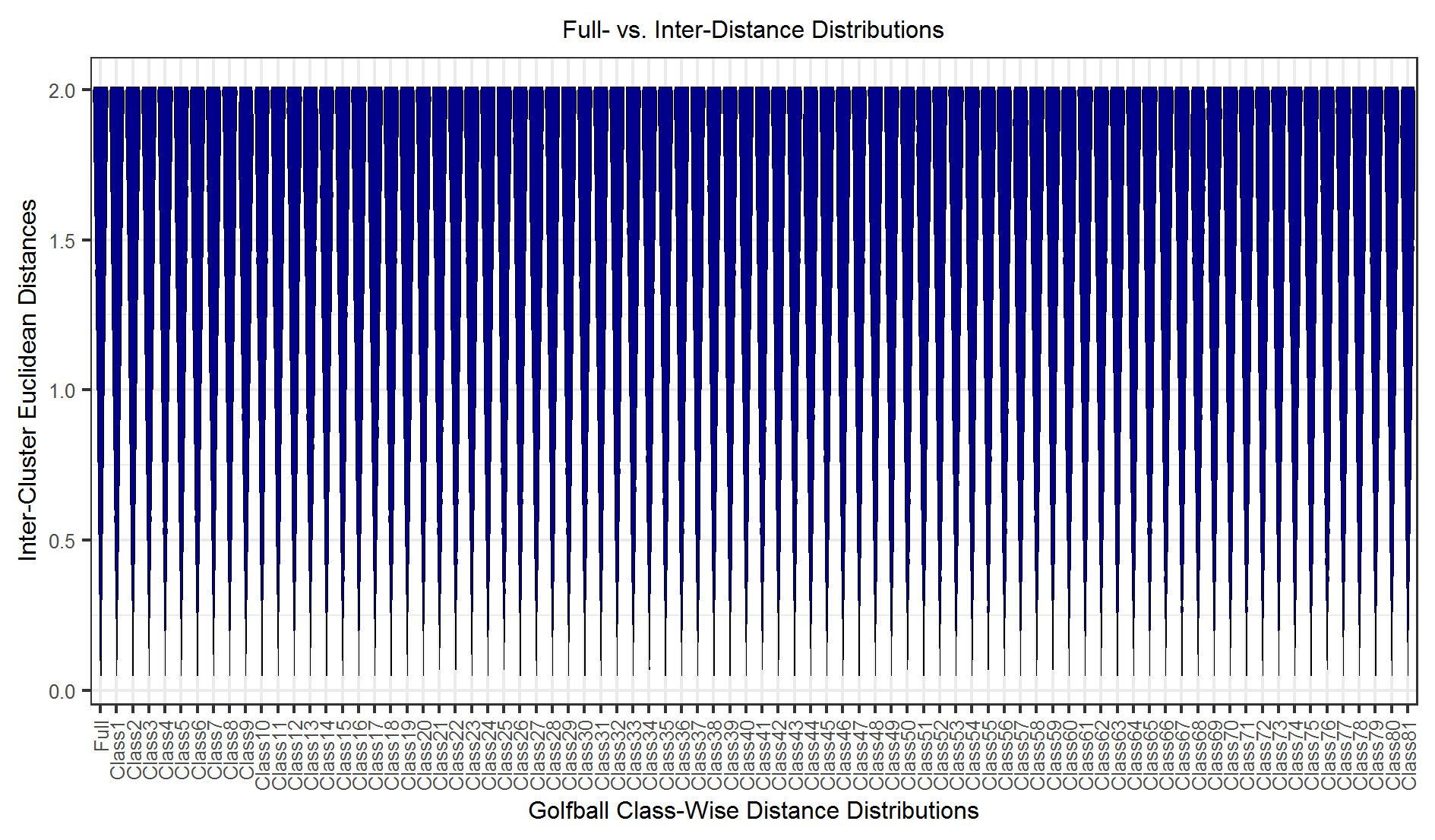


**Supplementary Fig. 5**. MD-plot of GolfBalls' Euclidean distance distribution and inter-cluster distances defined by SOM clustering for a Davies–Bouldin index^11^ of 0.83. No cluster structure is given; the inter-cluster distance distributions (right) have a large variance similar to that of the full distance distribution (first MD-plot on the left).


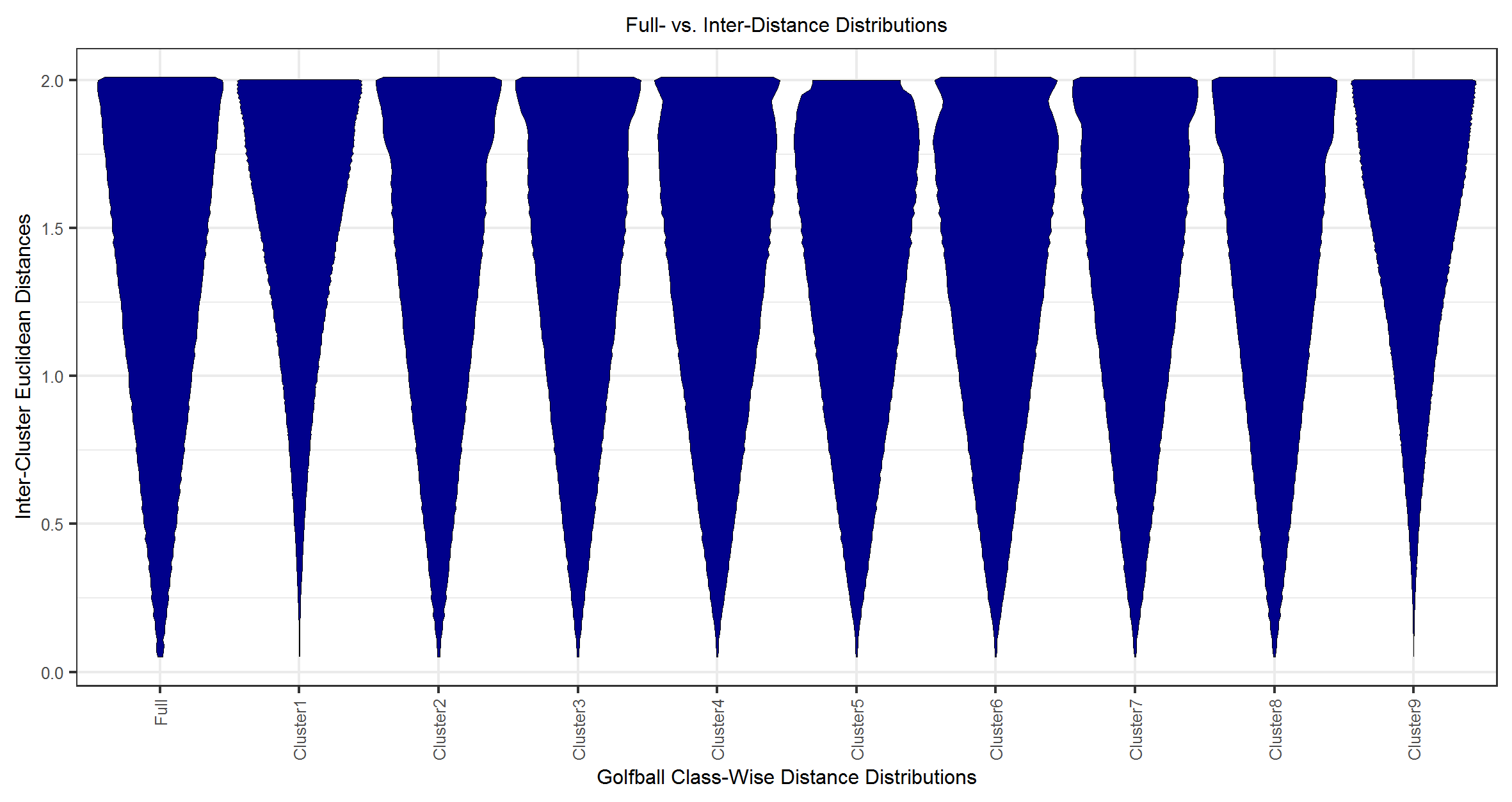


**Supplementary Fig. 6**. MD-plot of GolfBalls' Euclidean distance distribution and inter-cluster distances defined by SOM clustering for a Davies–Bouldin index^11^ of 11.8. No cluster structure is given; the inter-cluster distance distributions (right plot) have a large variance similar to the full distance distribution (first MD-plot on the left).


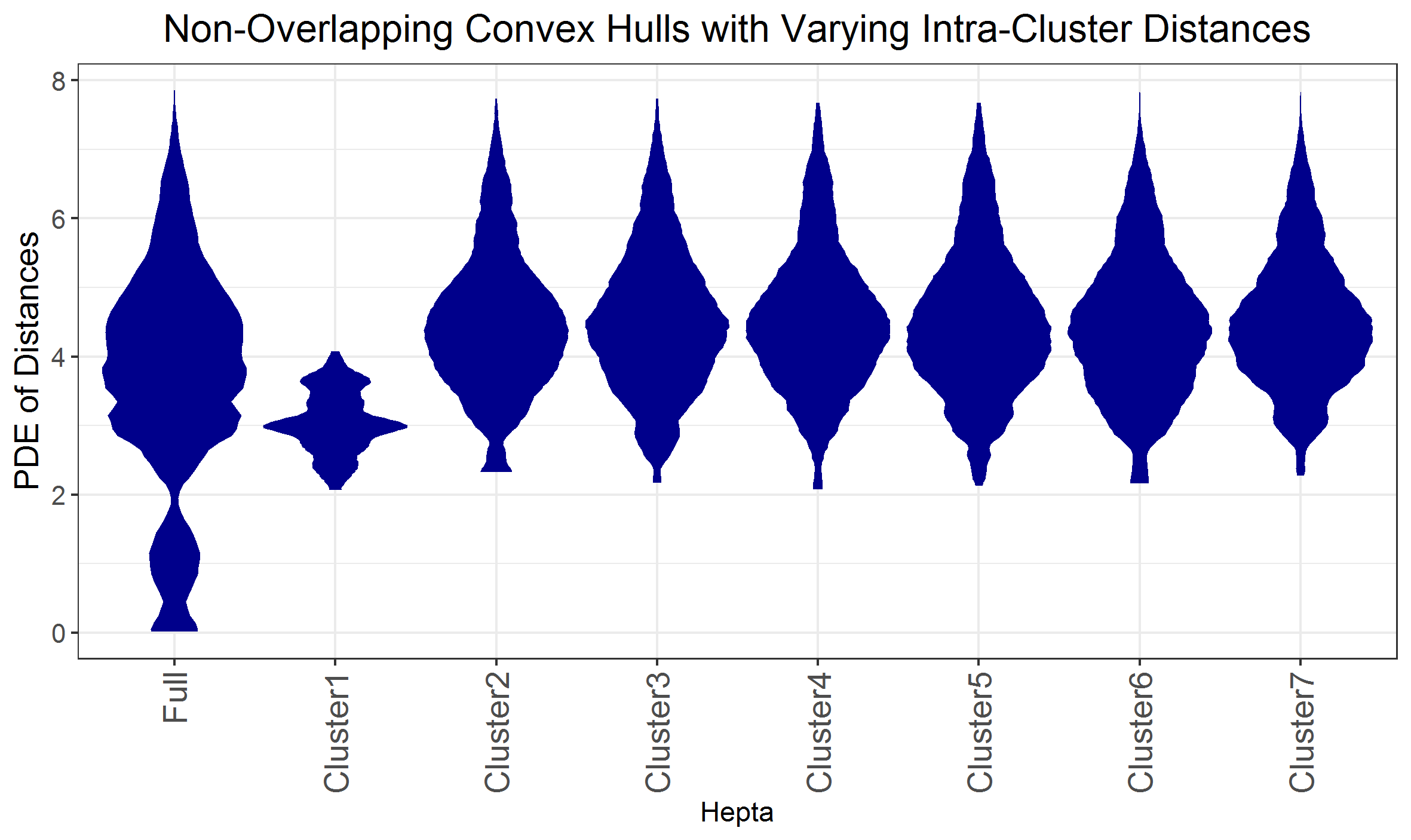


**Supplementary** **Fig. 7**. MD-plot of the data structures with non-overlapping convex hulls of the Hepta data set with varying intra-cluster distances. Full depicts the distribution of the distance matrix of all datapoints.


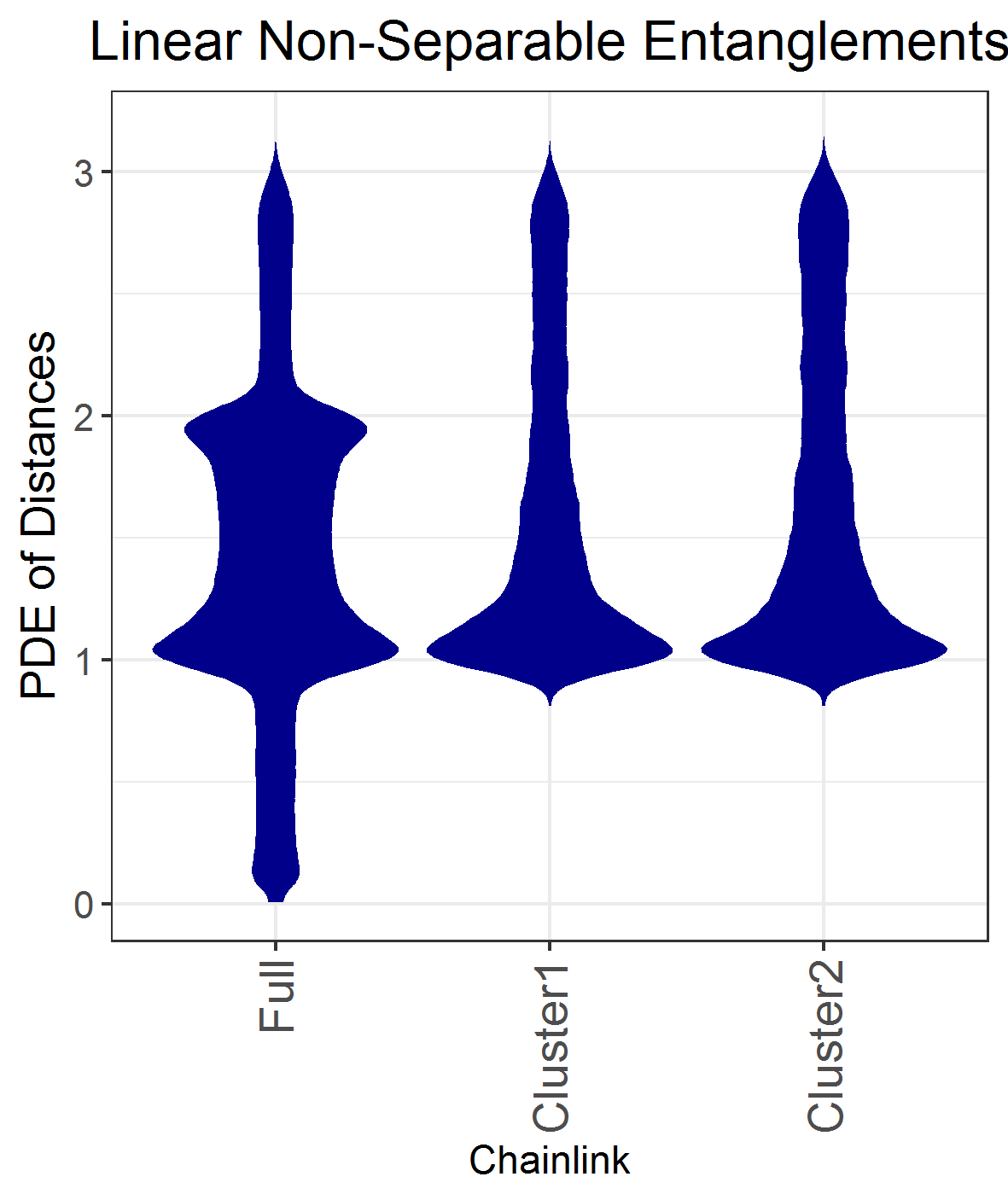

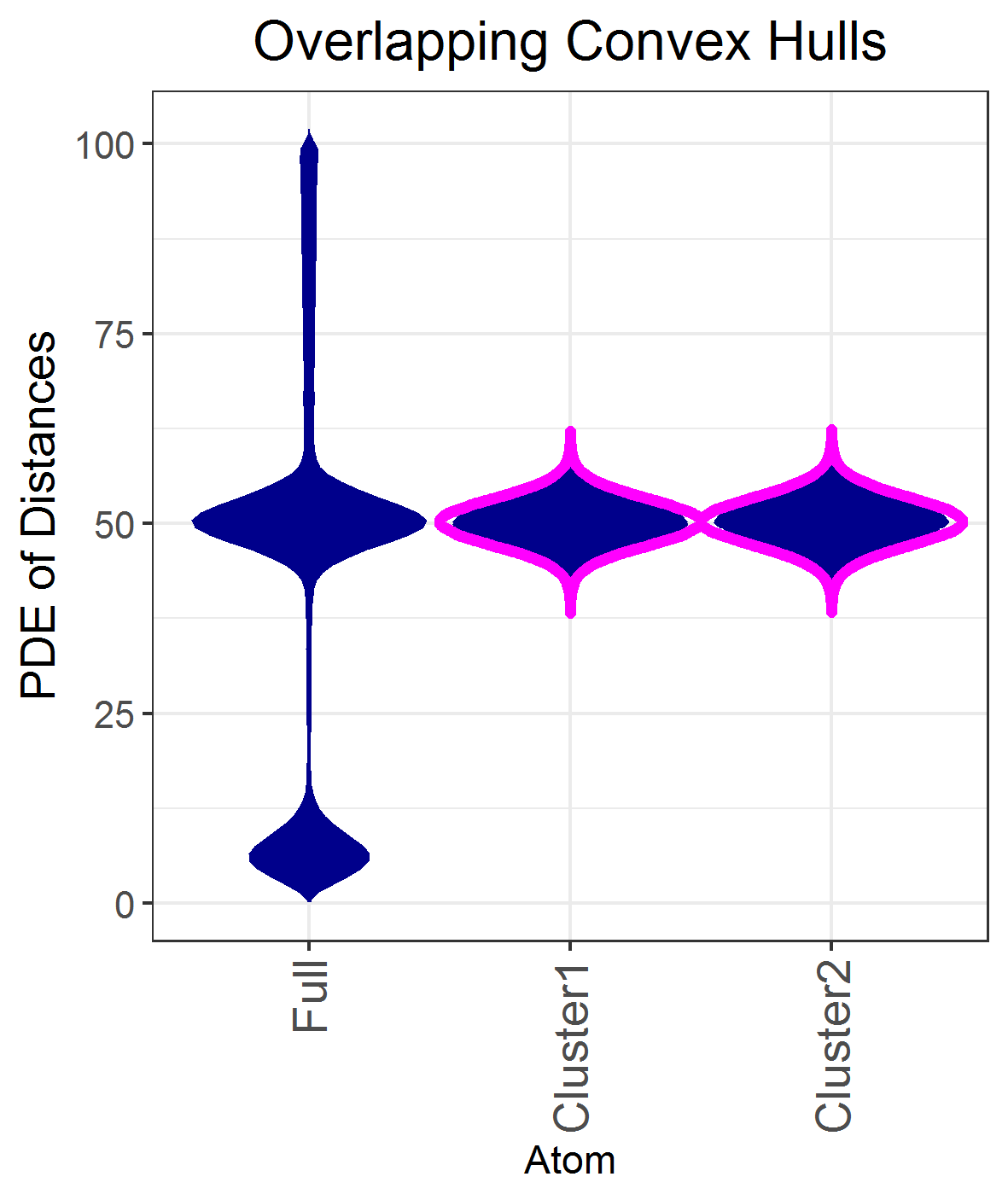


**Supplementary** **Fig. 8**. MD-plot of the data structures with linearly non-separable entanglements of the Chainlink data set (left) and overlapping convex hulls (right). Full depicts the distribution of the distance matrix of all datapoints.


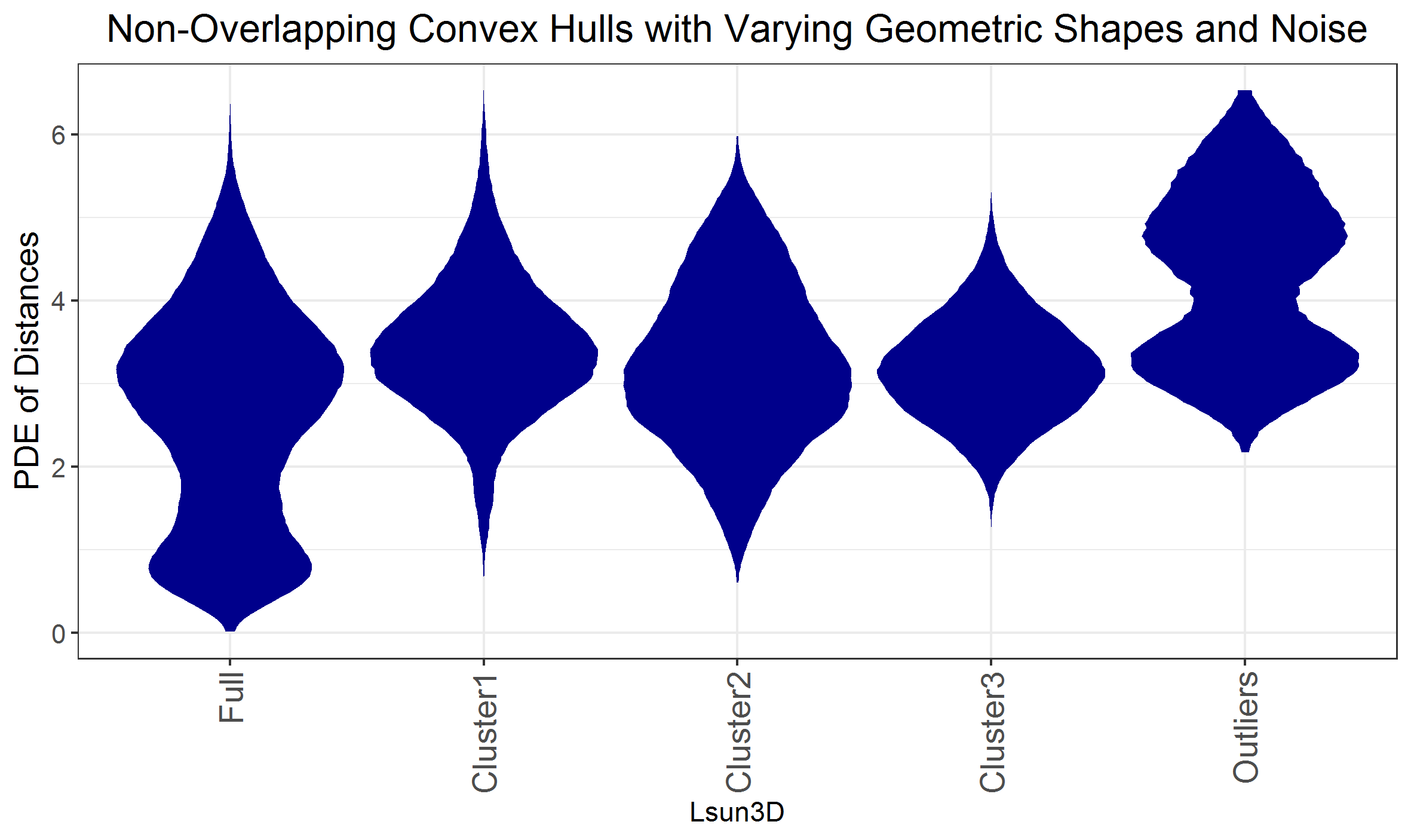


**Supplementary Fig. 9**. MD-plot of the data structures with non-overlapping convex hulls with varying geometric shapes and noise of the Lsun3D data set. Full depicts the distribution of the distance matrix of all datapoints.

***SI C: Unstable Clustering Solution of the Cancer Dataset***


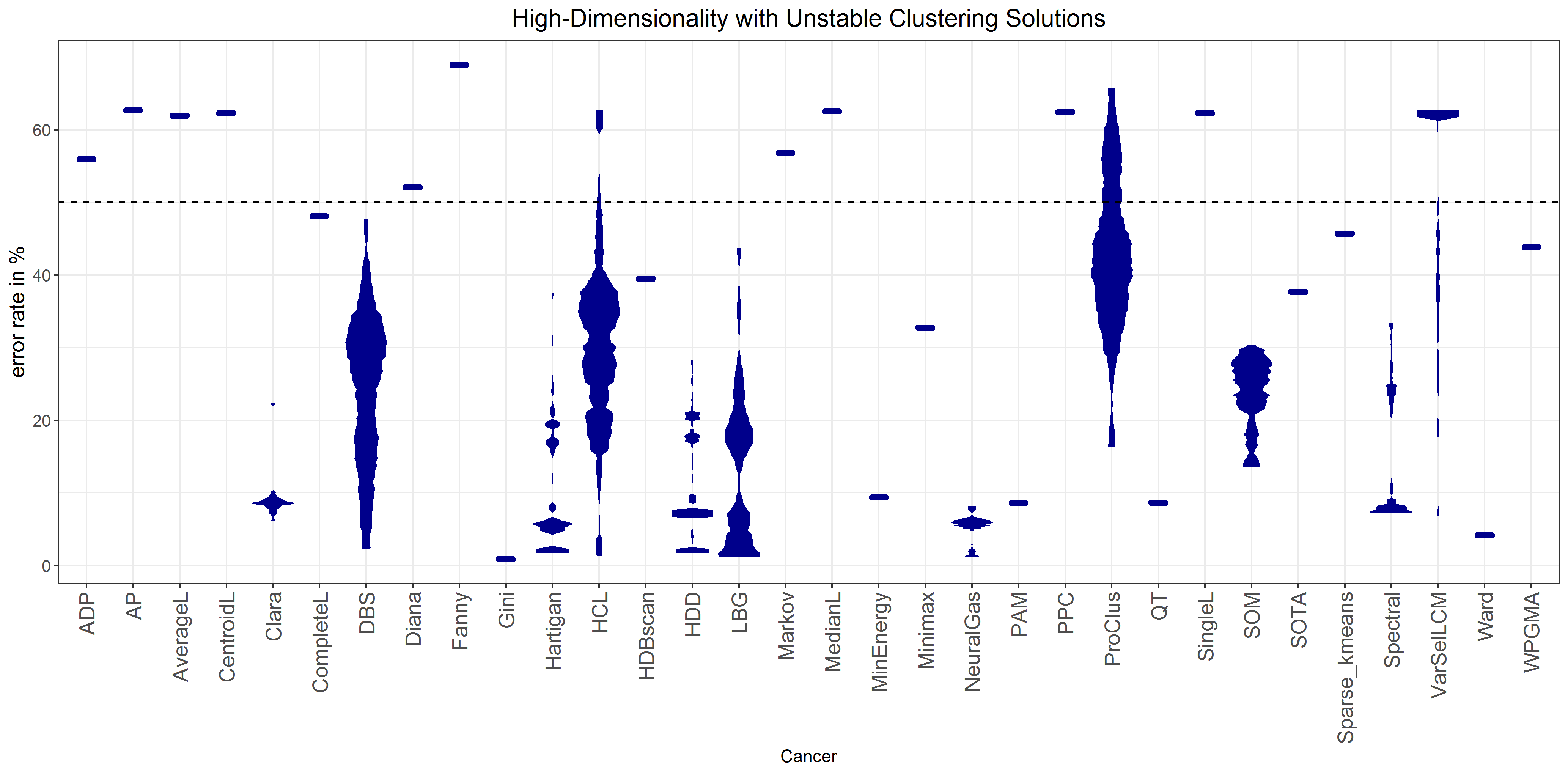

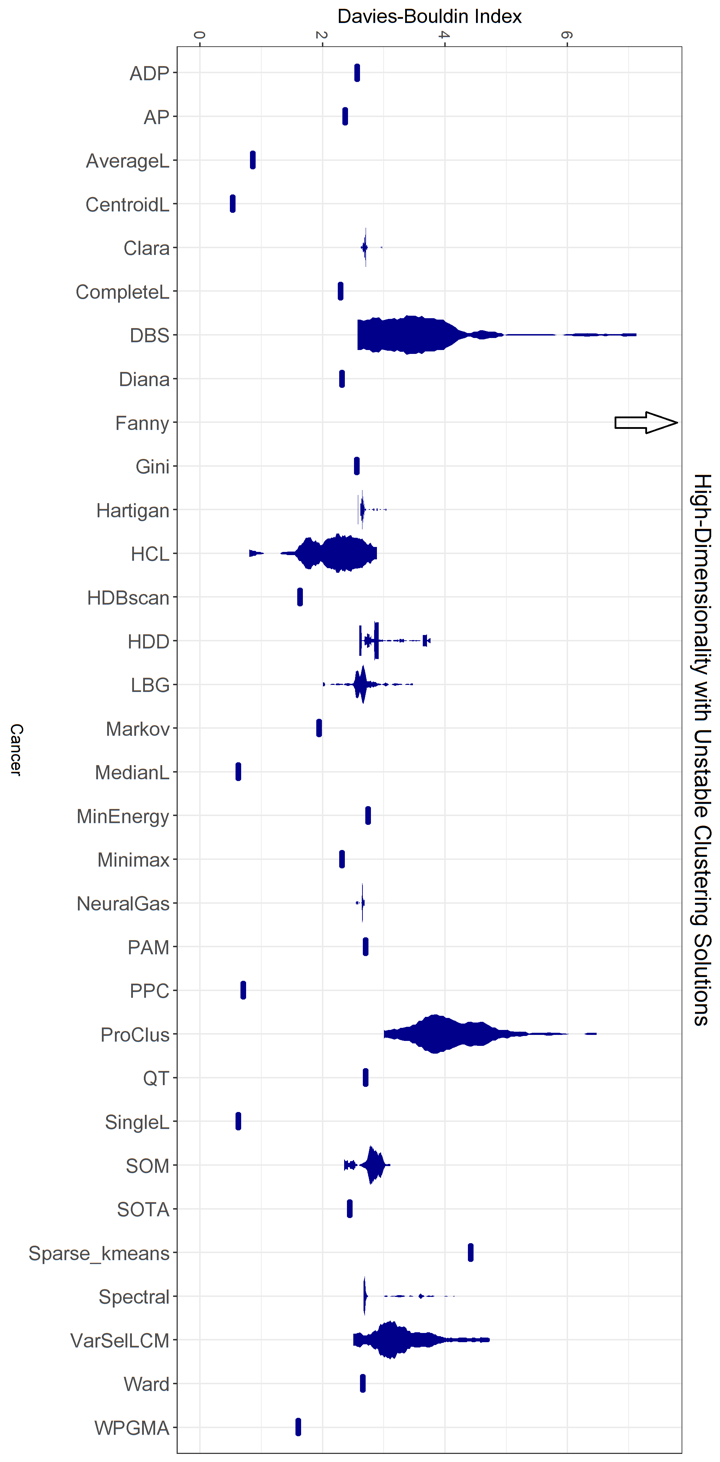


**Supplementary** **Fig. 10.** MD-plot of error rate (left) and Davies–Bouldin index (right) for 120 trials per algorithm for 32 clustering algorithms calculated for the cancer dataset. Distance-based structures based on this ground truth cannot be reproduced with high stability. The chance level is shown by the dotted line at 50%. The choice of an algorithm by the Davies–Bouldin index would lead to the selection of CentroidL, MedianL, SingleL, or PPC, whereas using the ground truth shows that Gini can reproduce the structures, DBS, Hartigan, HDD, LBG or NeuralGas can sometimes reproduce the structures; Ward has a small bias. Note that Fanny clustering has always the value of 21,1 for the Davies-Bouldin which is indicated by an arrow in this figure. The results for Clustvarsel, CrossEntropyC, ModelBased, mvnpEM, npEM, Orclus, RobustTrimmedC, SparseH and Spectrum could not be computed.

***SI D: MD plots Reveals Biases and the Various States of Probability***


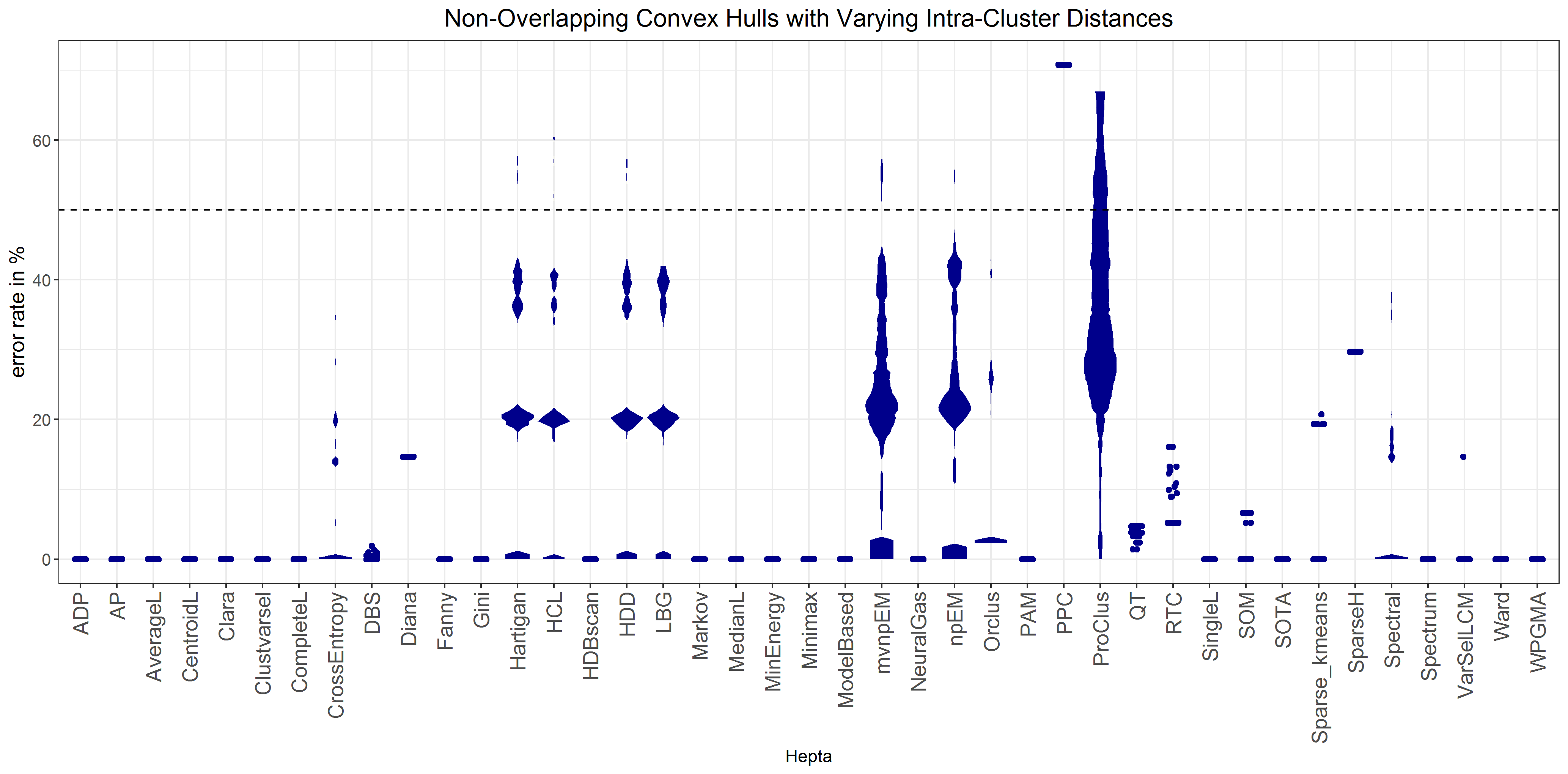


**Supplementary** **Fig. 11.** MD plot of the error rates for 120 trials per algorithm for 41 clustering algorithms is calculated for the Hepta dataset. Non-overlapping convex hulls of spherical shape with varying intra-cluster distances are the best case for high-dimensional data and, hence, should provide a simple task for clustering algorithms. The chance level is a dotted line at 50%. Different states of probability are visible for CrossEntropy, Hartigan, HCL, HDD, LBG , mvnpEM, npEM, Orclus, ProClus, RTC, SOM, Sparse k-means, and Spectral clustering. Contrary to insights from the literature (Duda et al., 2001; Handl et al., 2005; Hennig, 2015; Mirkin, 2005; Theodoridis & Koutroumbas, 2009)^12-16^, k-means methods are in this case unable to reproduce a spherical clustering structure in every trial because of the varying intra-cluster distances.


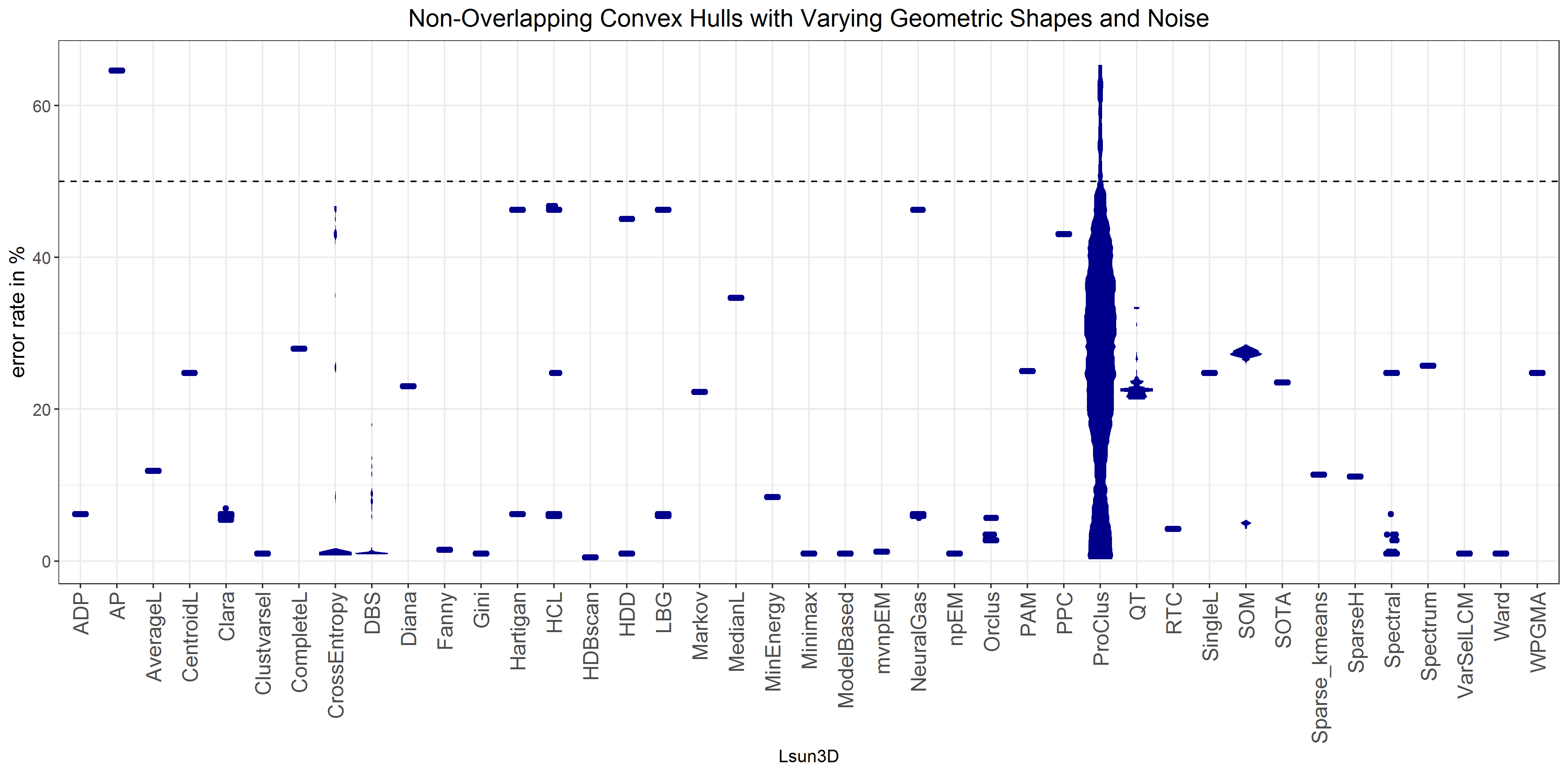


**Supplementary** **Fig. 12**. MD-plot of the error rates for 120 trials per algorithm for 41 clustering algorithms calculated for the Lsun3D dataset. Varying geometric shapes and outliers in the structures are not easy for conventional clustering algorithms to tackle. The chance level is shown by the dotted line at 50%. Clustvarsel, Gini, HDBSCAN, Minimax ModelBased, mvnEM, npEM, VarSelLCM and Ward are reasonably robust against outliers in the low-dimensional case. DBS, CrossEntropy, Fanny, HDD, Orclus and Spectral have some variance. In total, 25 out of the 41 clustering algorithms fail on this task.


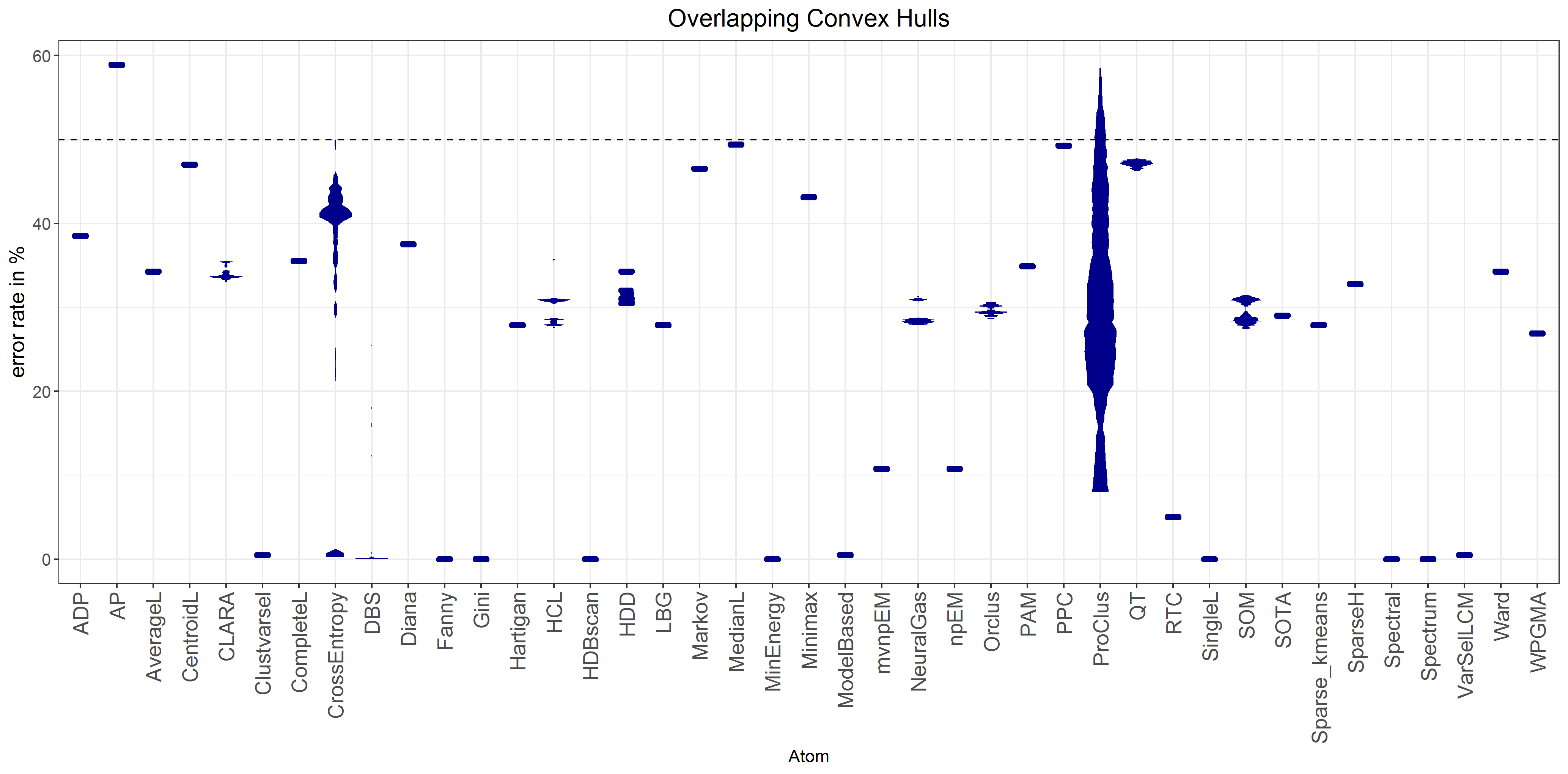


**Supplementary** **Fig. 13**. MD-plot of the error rates for 120 trials per algorithm for 41 clustering algorithms calculated for the Atom dataset. Entirely overlapping complex hulls are challenging for typical clustering algorithms. In total, 29 out of the 41 algorithms completely fail at this task. The chance level is shown by the dotted line at 50%.


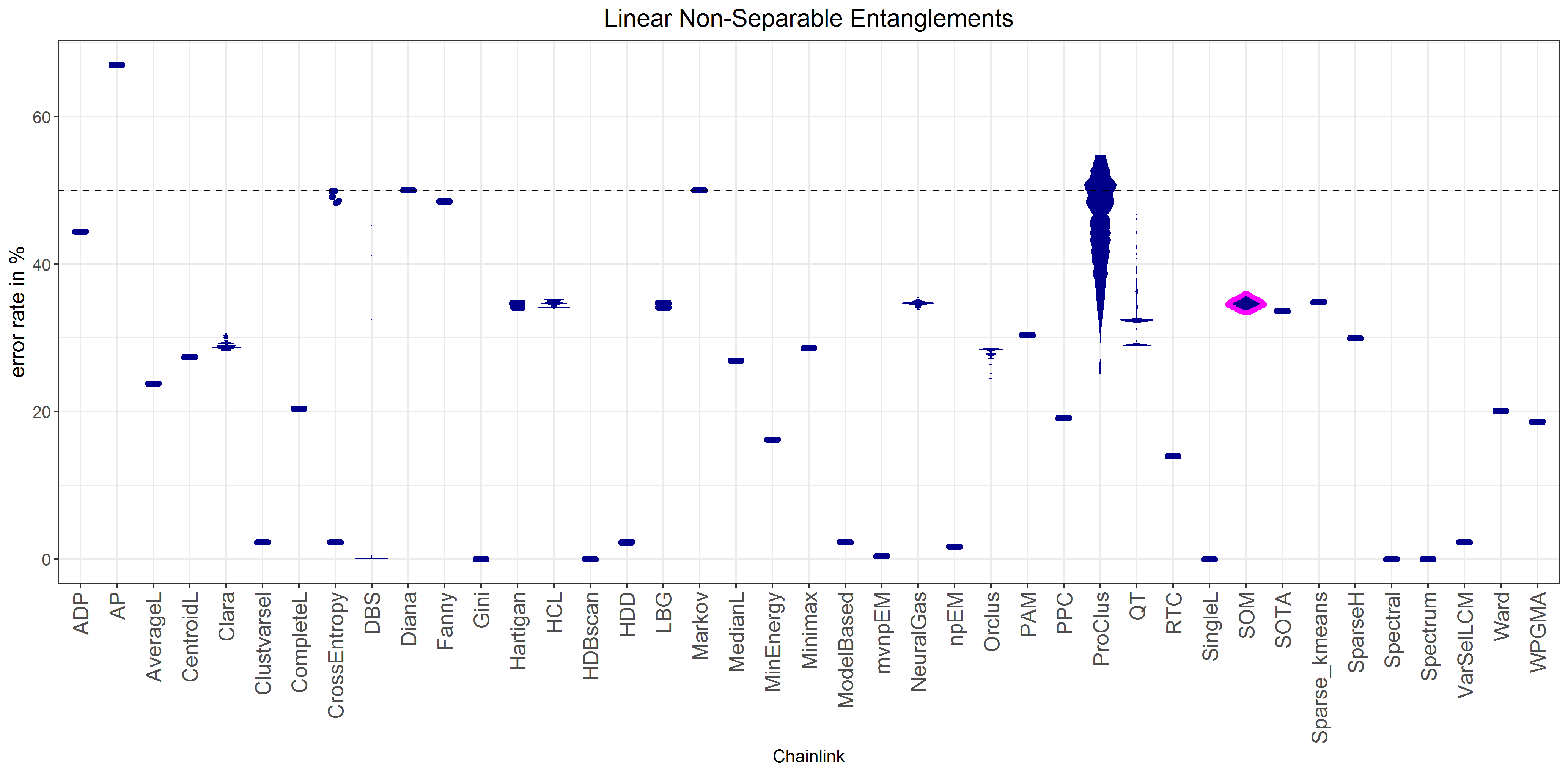


**Supplementary** **Fig. 14**. MD-plot of the error rates for 120 trials per algorithm for 41 clustering algorithms calculated for the Chainlink dataset. Finding non-linearly separable entanglements is the most difficult task to tackle in high-dimensional data. The chance level is shown by the dotted line at 50%. Only DBS, Gini, mvnpEM, HDBSCAN, Spectral, SingleL, and Spectrum clustering reproduce these structures. Clustvarsel, Crossentropy, ModelBased, npEM and VarSekKCN clustering has a small bias and DBS four outliers. In total, 29 out of the 41 clustering algorithms fail in this task

***SI: E Further Leukaemia and Cancer Evaluations***


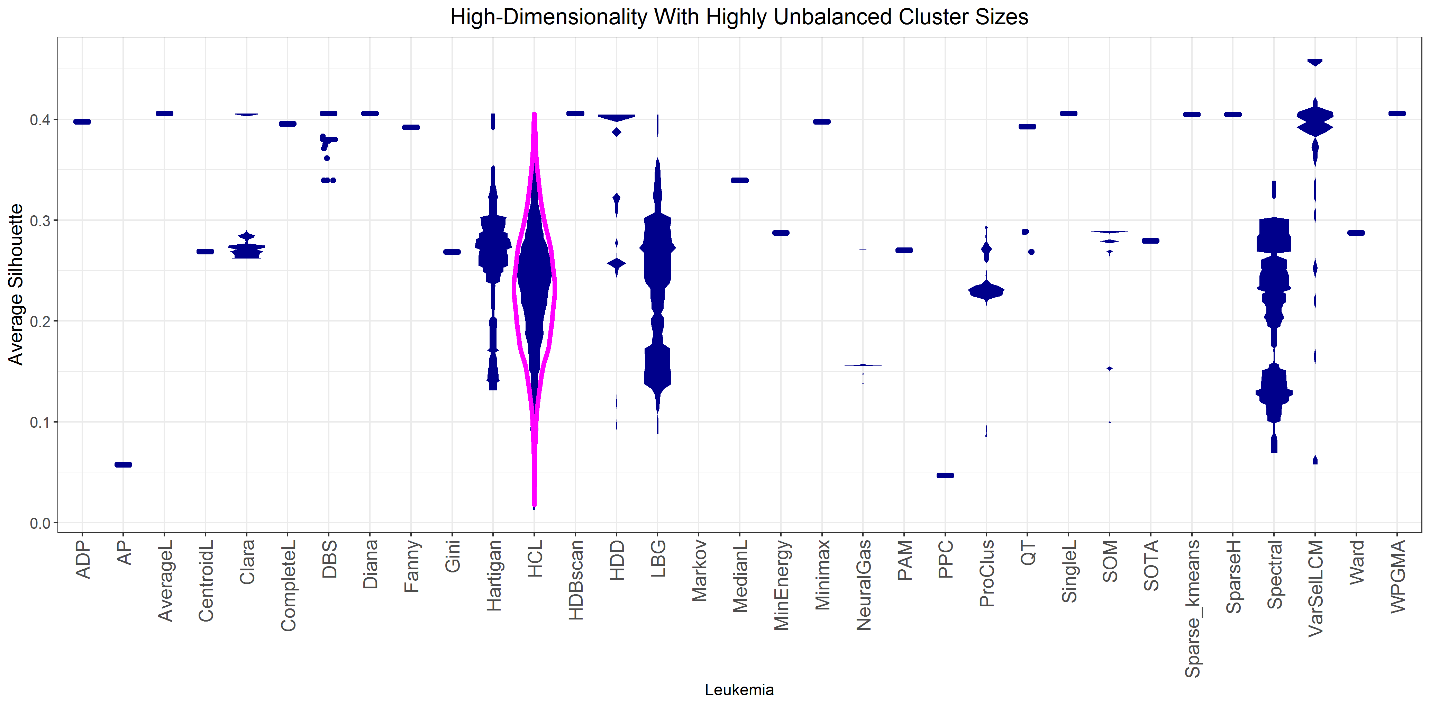


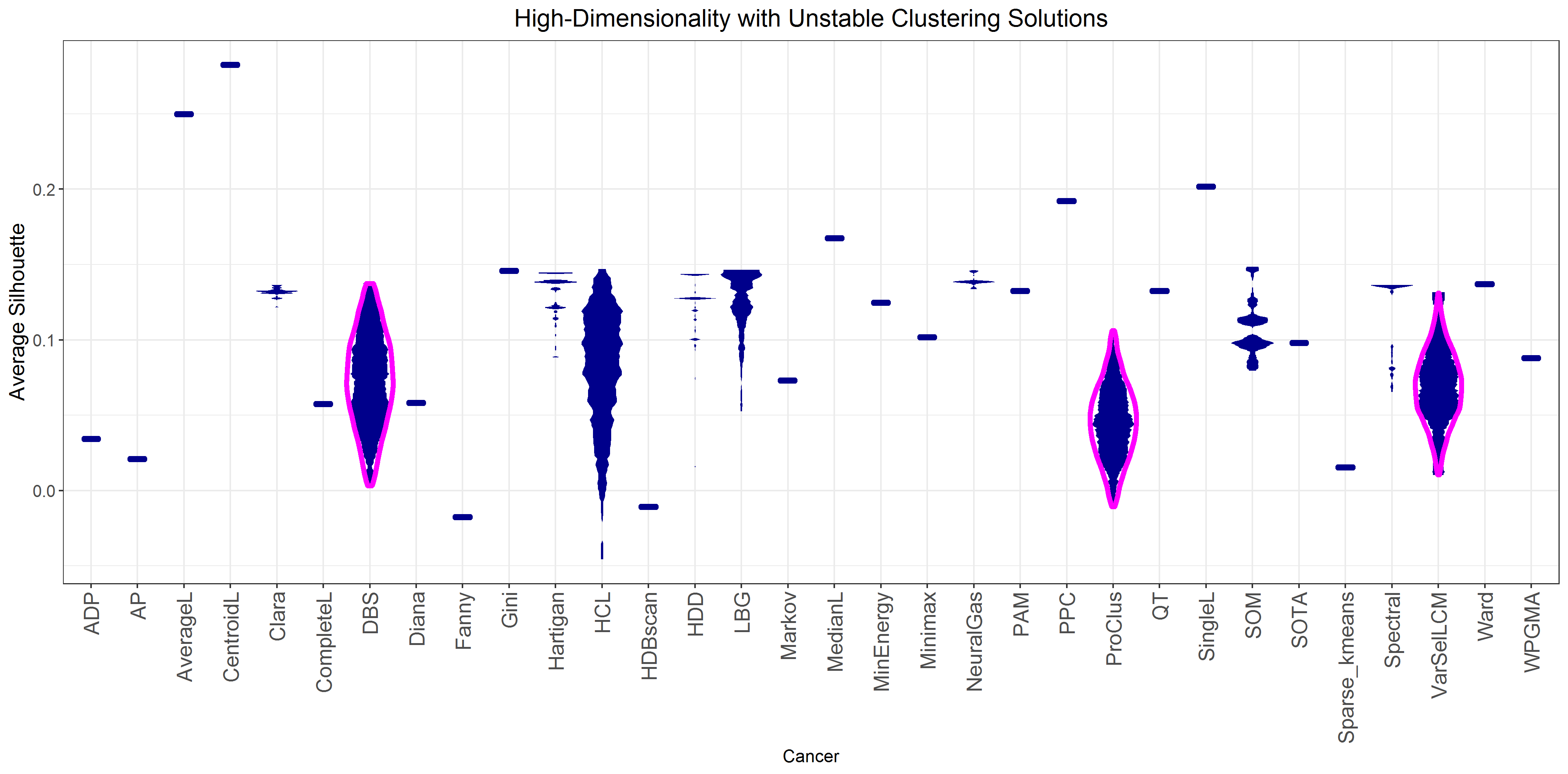


**Supplementary Fig. 15**. MD-plot of the silhouette index^17^ of the leukaemia (top) and cancer (bottom) datasets yields misleading results. Typically, values below 0.25 mean no structures and below 0.5 lead to the assumption that the structure is weak and can be disregarded^17^. However, SI A presents a clear distance-based cluster structure. In the case of the leukaemia dataset, VarSelLCM would be selected, and in the case of cancer, the CentroidL algorithm would be selected. Note that, for the Markov clustering of the leukaemia dataset the silhouette index cannot be calculated because only one cluster is given.


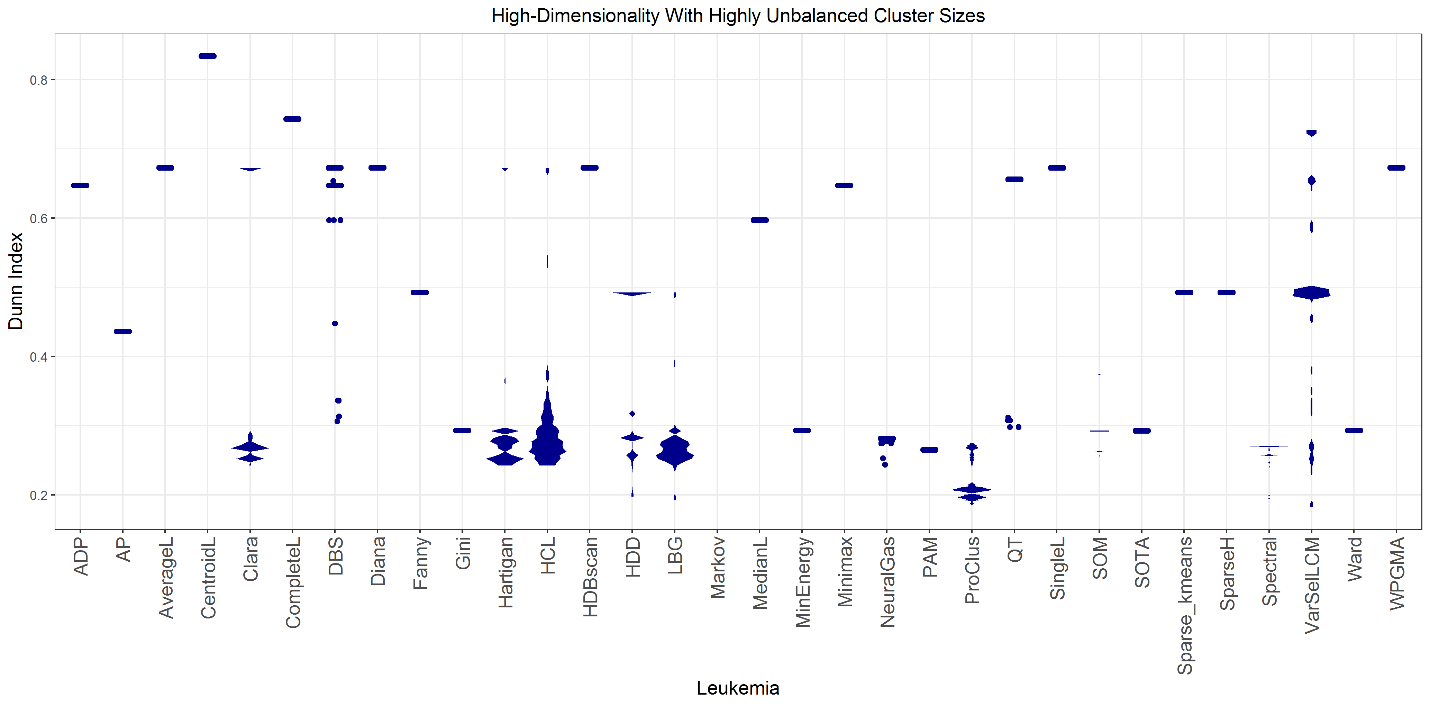


**
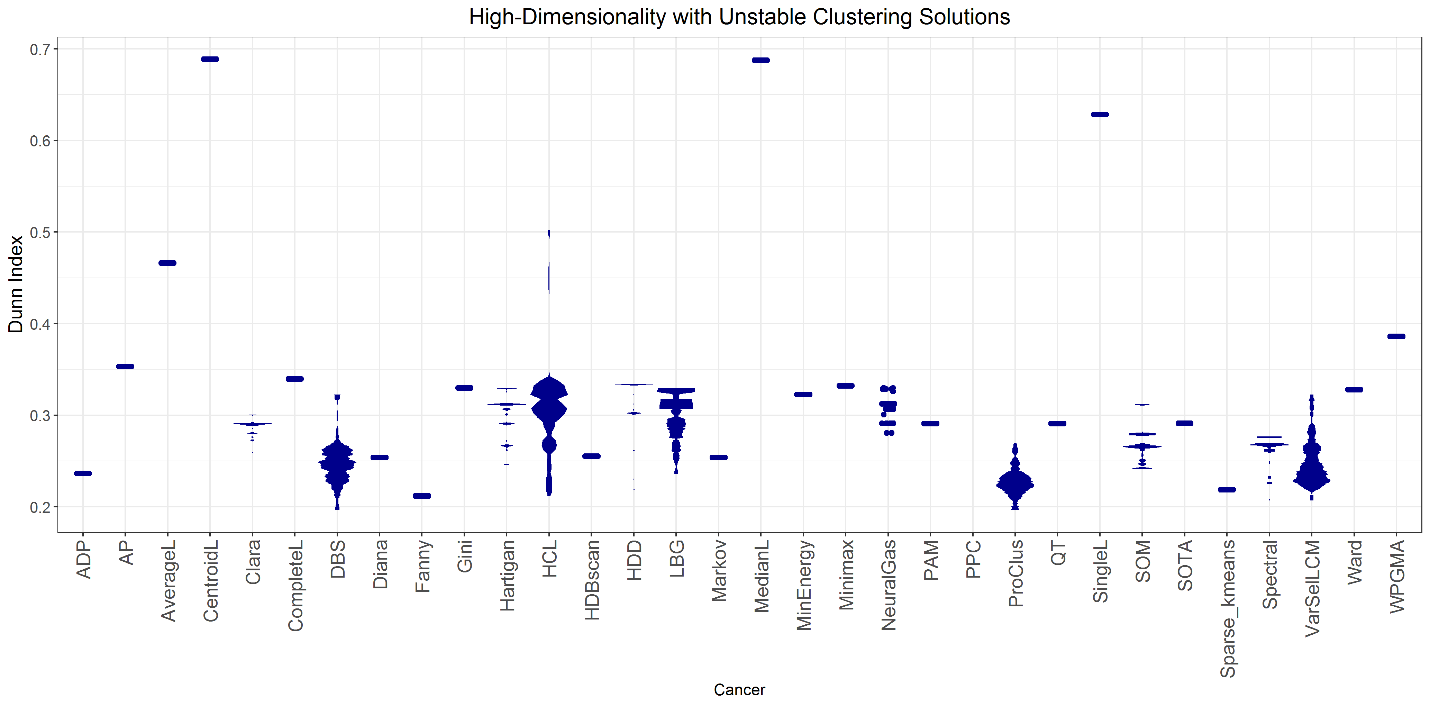
**

**Supplementary Fig. 16**. MD-plot of the Dunn index^18^ for the leukaemia (top) and cancer (bottom) datasets yields misleading results. In the case of the leukaemia dataset, CentroidL clustering would be selected, and in the case of the cancer dataset, CentroidL or MedianL would be select. It should be noted that Dunn suggests values above 1 as appropriate for clustering^18^. Note that, for the Markov clustering of leukaemia dataset the Dunn index cannot be calculated because only one cluster is given and for the PPC clustering of the cancer dataset all data points are in cluster one besides one point in which case also the Dunn index cannot be calculated.

***SI F: Clustering Algorithms Used in Benchmarking***

Supplementary Table 1 presents the algorithms selected for this work based on the R package ‘FCPS’ on CRAN^19^ (further extended after publishing^18^), and whether the variance of the results can be expected based in the results of this work. It should be noted that the authors^18^ were unable to implement transitivity clustering^[[1]](#footnote-1)20^.

**Supplementary Table 1**. Overview of the clustering algorithms used in benchmarking, their abbreviations, whether they require the number of clusters (NOC) to be set, whether they accept data or distances as input, the number of non-default parameters N, whether the variance of the results can be expected, and the authors of the algorithms.

| **Abbreviations** | **Algorithm** | **NOC** | **DOD** | ***N*** | **Variance** | **Authors** |
| --- | --- | --- | --- | --- | --- | --- |
| ADP | Adaptive Density Peak | Yes | Data | 1 | Yes | Rodriguez and Laio^21^ and Wang and Xu^22^ |
| AP | Affinity Propagation | No | Both | 0 | No | Bodenhofer et al.^23^ and Frey and Dueck^24^ |
| AverageL | Average Linkage | Yes | Both | 1 | No | Sokol and Michener^25^ |
| CentroidL | Centroid Linkage | Yes | Both | 1 | No | Sokol and Michener^25^ |
| Clara | Large Application Clustering | Yes | Data | 1 | Yes | Rousseeuw and Kaufman^26^ |
| Clustvarsel | Model-Based Clustering with variable selection | Yes | Data | 1 | Yes | Scrucca and Raftery^27^ |
| CompleteL | Complete Linkage | Yes | Both | 1 | No | Defays^28^ and Lance and Williams^29^ |
| CrossEntropy | Cross-Entropy Clustering | Yes | Data | 1 | Yes | Spurek et al.^30^ and Tabor and Spurek^31^ |
| DBS | Databionic Swarm | Yes | Both | 1 | Yes | Thrun and Ultsch^32^ |
| Diana | Divisive Analysis Clustering | Yes | Both | 1 | No | Rousseeuw and Kaufman^26^ |
| Fanny | Fuzzy Analysis Clustering | Yes | Both | 1 | No | Rousseeuw and Kaufman^26^ |
| Gini | Genie Clustering by the Gini Index | Yes | Both | 1 | Yes | Gagolewski et al.^33^ |
| Hartigan | Hartigan k-Means | Yes | Data | 1 | Yes | Hartigan and Wong^34^ |
| HCL | Hard Competitive Learning (Online Update) | Yes | Data | 1 | Yes | Ripley^35^ |
| HDBSCAN | Hierarchical DBSCAN | No^2^ | Both | 0 | No | Campello et al.^36^ and Hahsler et al.^37^ |
| HDD | High-Dimensional Data Clustering | Yes | Data | 1 | Yes | Bouveyron et al^38^ |
| LBG | LBG k-means | Yes | Data | 1 | Yes | Linde et al.^39^ |
| Markov | Markov Clustering | No | Both | 1 | No | Van Dongen^40^ |
| MedianL | Median Linkage | Yes | Both | 1 | No | Everitt et al.^41^ and Lance and Williams^42^ |
| MinEnergy | Minimal Energy | Yes | Both | 1 | No | Szekely and Rizzo^43^ |
| Minimax | Minimax Linkage | Yes | Both | 1 | Yes | Azzalini and Torelli^44^ |
| ModelBased | Model-Based Clustering without variable selection | Yes | Data | 1 | No | Fraley and Raftery^45^ |
| mvnpEM | Mixture of Gaussians Clustering | Yes | Data | 1 | Yes | Chauveau and Hoang^46^ |
| Neural Gas | Neural Gas | Yes | Data | 1 | Yes | Martinetz et al.^47^ |
| npEM | Mixture of Gaussians Clustering | Yes | Data | 1 | Yes | Benaglia et al.^48^ |
| Orclus | Orclus Subspace Clustering | Yes | Data | 1 | Yes | Aggarwal and Yu^49^ |
| PAM | Partitioning Around Medoids | Yes | Both | 1 | Yes | Rousseeuw and Kaufman^26^ |
| ProClus | ProClus Subspace Clustering | Yes | Data | 1 | Yes | Aggarwal et al.^50^ |
| PPC | Projection Pursuit Clustering | Yes | Data | 1 | No | Hofmeyr and Pavlidis^51^ |
| QT | Stochastic Quality Clustering | No | Data | 1 | Yes | Heyer et al.^52^ |
| RTC | Robust Trimmed Clustering | Yes | Data | 2 | Yes | Fritz et al.^53^ and García-Escudero et al.^54^ |
| SingleL | Single Linkage | Yes | Both | 1 | No | Florek et al.^55^ |
| SOM | Self-Organizing Maps | Yes | Data | 2 | Yes | Wehrens and Buydens^56^ |
| SOTA | Self-organizing Tree Algorithm | Yes | Data | 1 | No | Herrero et al.^57^ |
| Sparse  k-means | SparseClustering | Yes | Data | 1 | Yes | Witten and Tibshirani^58^ |
| SparseH | SparseClustering | Yes | Both | 1 | No | Witten and Tibshirani^58^ |
| Spectral | Spectral clustering | Yes | Data | 1 | Yes | Ng et al.^59^ |
| Spectrum | Fast Adaptive Spectral Clustering | Yes^3^ | Data | 0-1 | No | John et al.^60^ |
| VarSelLCM | Model-Based Clustering with variable selection | Yes | Data | 1 | Yes | Marbac and Sedki^61-63^ |
| Ward | Ward | Yes | Both | 1 | No | Murtagh and Legendre^64^ and Ward Jr.^65^ |
| WPGMA | McQuitty Clustering | Yes | Both | 1 | No | McQuitty^66^ |

1. Thrun, M. C. The Exploitation of Distance Distributions for Clustering. *International Journal of Computational Intelligence and Applications* 20, 2150016 (2021).

2. Haferlach, T. et al. Clinical utility of microarray-based gene expression profiling in the diagnosis and subclassification of leukemia: report from the International microarray innovations in leukemia study group. *J. Clin. Oncol.* 28, 2529-2537 (2010).

3. Lo-Coco, F. et al. Retinoic acid and arsenic trioxide for acute promyelocytic leukemia. *N. Engl. J. Med.* 369, 111-121 (2013).

4. Venna, J., Peltonen, J., Nybo, K., Aidos, H. & Kaski, S. Information retrieval perspective to nonlinear dimensionality reduction for data visualization. *J. Mach. Learn. Res.* 11, 451-490 (2010).

5. Ultsch, A. & Thrun, M. C. in *12th International Workshop on Self-Organizing Maps and Learning Vector Quantization, Clustering and Data Visualization (WSOM)* (Eds. Cottrell, M.) 1-5 (IEEE, Nany, France, 2017).

6. Thrun, M. C. *Projection Based Clustering through Self-Organization and Swarm Intelligence* (Springer, Heidelberg, 2018).

7. Ultsch, A. & Lötsch, J. Machine-learned cluster identification in high-dimensional data. *J. Biomed. Inform.* 66, 95-104 (2017).

8. Thrun, M. C. & Ultsch, A. Uncovering High-Dimensional Structures of Projections from Dimensionality Reduction Methods. *MethodsX* 7, 101093 (2020).

9. Weinstein, J. N. et al. The cancer genome atlas pan-cancer analysis project. *Nat. Genet.* 45, 1113-1120 (2013).

10. Lichman, M. *UCI Machine Learning Repository* (University of California, School of Information and Computer Science, Irvine, CA, 2013).

11. Davies, D. L. & Bouldin, D. W. A cluster separation measure. *IEEE Trans. Pattern Anal. Mach. Intell.* 1, 224-227 (1979).

12. Duda, R. O., Hart, P. E. & Stork, D. G. *Pattern Classification* (John Wiley & Sons, Ney York, NY, 2001).

13. Handl, J., Knowles, J. & Kell, D. B. Computational cluster validation in post-genomic data analysis. *Bioinformatics* 21, 3201-3212 (2005).

14. Hennig, C., Meila, M., Murtagh, F. & Rocci, R. *Handbook of cluster analysis* (Chapman & Hall/CRC Press, New York, NY, 2015).

15. Mirkin, B. G. *Clustering: A Data Recovery Approach* (Chapnman & Hall/CRC, Boca Raton, FL, 2005).

16. Theodoridis, S. & Koutroumbas, K. *Pattern Recognition* (Elsevier, Canada, 2009).

17. Rousseeuw, P. J. Silhouettes: a graphical aid to the interpretation and validation of cluster analysis. *J. Comput. Appl. Math.* 20, 53-65 (1987).

18. Dunn, J. C. Well-separated clusters and optimal fuzzy partitions. *J. Cybern.* 4, 95-104 (1974).

19. Thrun, M. C. & Stier, Q. Fundamental Clustering Algorithms Suite *SoftwareX* 13, 100642 (2021).

20. Wittkop, T. et al. Partitioning biological data with transitivity clustering. *Nat. Methods* 7, 419 (2010).

21. Rodriguez, A. & Laio, A. Clustering by fast search and find of density peaks. *Science* 344, 1492-1496 (2014).

22. Wang, X.-F. & Xu, Y. Fast clustering using adaptive density peak detection. *Stat. Methods Med. Res.* 26, 2800-2811 (2017).

23. Bodenhofer, U., Kothmeier, A. & Hochreiter, S. APCluster: an R package for affinity propagation clustering. *Bioinformatics* 27, 2463-2464 (2011).

24. Frey, B. J. & Dueck, D. Clustering by passing messages between data points. *science* 315, 972-976 (2007).

25. Sokol, R. & Michener, C. A statistical method for evaluating systematic relationships. *Kans. Sci. Bull.* 28, 1409-1438 (1958).

26. Rousseeuw, P. J. & Kaufman, L. *Finding Groups in Data* (John Wiley & Sons Inc, Belgium, 1990).

27. Scrucca, L. & Raftery, A. E. clustvarsel: a package implementing variable selection for Gaussian model-based clustering in R. *Journal of Statistical Software* 84, (2018).

28. Defays, D. An efficient algorithm for a complete link method. *Comput. J.* 20, 364-366 (1977).

29. Lance, G. N. & Williams, W. T. A general theory of classificatory sorting strategies: 1. Hierarchical systems. *Comput. J.* 9, 373-380 (1967).

30. Spurek, P., Kamieniecki, K., Tabor, J., Misztal, K. & Śmieja, M. R package cec. *Neurocomputing* 237, 410-413 (2017).

31. Tabor, J. & Spurek, P. Cross-entropy clustering. *Pattern Recognit.* 47, 3046-3059 (2014).

32. Thrun, M. C. & Ultsch, A. Swarm intelligence for self-organized clustering. *Artif. Intell.* 290, 103237 (2020).

33. Gagolewski, M., Bartoszuk, M. & Cena, A. Genie: a new, fast, and outlier-resistant hierarchical clustering algorithm. *Inf. Sci.* 363, 8-23 (2016).

34. Hartigan, J. A. & Wong, M. A. Algorithm AS 136: a k-means clustering algorithm. *J. R. Stat. Soc. C (Appl. Stat.)* 28, 100-108 (1979).

35. Ripley, B. D. *Pattern recognition and neural networks* (Cambridge university press, 2007).

36. Campello, R. J., Moulavi, D., Zimek, A. & Sander, J. Hierarchical density estimates for data clustering, visualization, and outlier detection. *ACM Trans. Knowl. Discov. Data (TKDD)* 10, 1-51 (2015).

37. Hahsler, M., Piekenbrock, M. & Doran, D. dbscan: fast density-based clustering with r. *J. Stat. Softw.* 25, 409-416 (2019).

38. Bouveyron, C., Girard, S. & Schmid, C. High-dimensional data clustering. *Computational statistics & data analysis* 52, 502-519 (2007).

39. Linde, Y., Buzo, A. & Gray, R. An algorithm for vector quantizer design. *IEEE Trans. Commun.* 28, 84-95 (1980).

40. Van Dongen, S. M. *Graph Clustering by Flow Simulation* (Universiteit Utrecht, Utrecht, Netherlands, 2000).

41. Everitt, B. S., Landau, S., Leese, M. & Stahl, D. in *Cluster Analysis* (Eds. Everitt, B. S., Landau, S., Leese, M. & Stahl, D.) 71-110 (John Wiley & Sons, Hoboken, NJ, 2011).

42. Lance, G. N. & Williams, W. T. Computer programs for hierarchical polythetic classification (“similarity analyses”). *Comput. J.* 9, 60-64 (1966).

43. Szekely, G. J. & Rizzo, M. L. Hierarchical clustering via joint between-within distances: extending ward's minimum variance method. *J. Classif.* 22, 151-183 (2005).

44. Azzalini, A. & Torelli, N. Clustering via nonparametric density estimation. *Stat. Comput.* 17, 71-80 (2007).

45. Fraley, C. & Raftery, A. E. *MCLUST Version 3: An R Package for Normal Mixture Modeling and Model-Based Clustering. Technical Report No. 504* (Department of Statistics, University of Washington, Seattle, US, 2006).

46. Chauveau, D. & Hoang, V. T. L. Nonparametric mixture models with conditionally independent multivariate component densities. *Computational Statistics & Data Analysis* 103, 1-16 (2016).

47. Martinetz, T. M., Berkovich, S. G. & Schulten, K. J. 'Neural-gas' network for vector quantization and its application to time-series prediction. *IEEE Trans. Neural Netw.* 4, 558-569 (1993).

48. Benaglia, T., Chauveau, D. & Hunter, D. R. An EM-like algorithm for semi-and nonparametric estimation in multivariate mixtures. *Journal of Computational and Graphical Statistics* 18, 505-526 (2009).

49. Aggarwal, C. C. & Yu, P. S. in *Proceedings of the 2000 ACM SIGMOD international conference on Management of data* 70-81 2000).

50. Aggarwal, C. C., Wolf, J. L., Yu, P. S., Procopiuc, C. & Park, J. S. in *ACM SIGMOD International Conference on Management of Data* 61-72 (Association for Computing Machinery, Philadelphia, Pennsylvania, USA, 1999).

51. Hofmeyr, D. & Pavlidis, N. PPCI: an R package for cluster identification using projection pursuit. *R J.* 11, 152-170 (2019).

52. Heyer, L. J., Kruglyak, S. & Yooseph, S. Exploring expression data: identification and analysis of coexpressed genes. *Genome Res.* 9, 1106-1115 (1999).

53. Fritz, H., Garcıa-Escudero, L. A. & Mayo-Iscar, A. tclust: an R package for a trimming approach to cluster analysis. *J. Stat. Softw.* 47, 1-26 (2012).

54. García-Escudero, L. A., Gordaliza, A., Matrán, C. & Mayo-Iscar, A. A general trimming approach to robust cluster analysis. *Ann. Stat.* 36, 1324-1345 (2008).

55. Florek, K., Łukaszewicz, J., Perkal, J., Steinhaus, H. & Zubrzycki, S. in *Colloquium Mathematicae* 282-285 (Institute of Mathematics Polish Academy of Sciences, Poland, 1951).

56. Wehrens, R. & Buydens, L. M. Self-and super-organizing maps in R: the Kohonen package. *J. Stat. Softw.* 21, 1-19 (2007).

57. Herrero, J., Valencia, A. & Dopazo, J. A hierarchical unsupervised growing neural network for clustering gene expression patterns. *Bioinformatics* 17, 126-136 (2001).

58. Witten, D. M. & Tibshirani, R. A framework for feature selection in clustering. *Journal of the American Statistical Association* 105, 713-726 (2010).

59. Ng, A. Y., Jordan, M. I. & Weiss, Y. On spectral clustering: analysis and an algorithm. *Adv. Neural Inf. Process. Syst.* 2, 849-856 (2002).

60. John, C. R., Watson, D., Barnes, M. R., Pitzalis, C. & Lewis, M. J. Spectrum: Fast density-aware spectral clustering for single and multi-omic data. *Bioinformatics* 36, 1159-1166 (2020).

61. Marbac, M. & Sedki, M. Variable selection for model-based clustering using the integrated complete-data likelihood. *Statistics and Computing* 27, 1049-1063 (2017).

62. Marbac, M. & Sedki, M. VarSelLCM: an R/C++ package for variable selection in model-based clustering of mixed-data with missing values. *Bioinformatics* 35, 1255-1257 (2018).

63. Marbac, M., Sedki, M. & Patin, T. Variable selection for mixed data clustering: application in human population genomics. *Journal of Classification* 37, 124-142 (2020).

64. Murtagh, F. & Legendre, P. Ward’s hierarchical agglomerative clustering method: which algorithms implement Ward’s criterion? *J. Classif.* 31, 274-295 (2014).

65. Ward Jr, J. H. Hierarchical grouping to optimize an objective function. *J. Am. Stat. Assoc.* 58, 236-244 (1963).

66. McQuitty, L. L. Similarity analysis by reciprocal pairs for discrete and continuous data. *Educ. Psychol. Meas.* 26, 825-831 (1966).

1. an R implementation does not exist and the ClustEval link (<https://clusteval.sdu.dk/1/programs>) is broken [↑](#footnote-ref-1)
